# A comparison of replicative senescence and doxorubicin-induced premature senescence of vascular smooth muscle cells isolated from human aorta

**DOI:** 10.1007/s10522-013-9477-9

**Published:** 2013-11-16

**Authors:** Anna Bielak-Zmijewska, Maciej Wnuk, Dorota Przybylska, Wioleta Grabowska, Anna Lewinska, Olga Alster, Zbigniew Korwek, Anna Cmoch, Aleksander Myszka, Slawomir Pikula, Grazyna Mosieniak, Ewa Sikora

**Affiliations:** 1 Department of Biochemistry, Nencki Institute of Experimental Biology, Polish Academy of Sciences, Pasteur Str. 3, 02-093 Warsaw, Poland; 2 Department of Genetics, University of Rzeszow, Rzeszow, Poland; 3 Centre of Applied Biotechnology and Basic Sciences, University of Rzeszow, Kolbuszowa, Poland; 4 Department of Biochemistry and Cell Biology, University of Rzeszow, Rzeszow, Poland; 5 Present Address: Laboratory of Modeling in Biology and Medicine, Institute of Fundamental Technological Research, Polish Academy of Sciences, Pawińskiego 5B, Warsaw, Poland

**Keywords:** Senescence, VSMCs, Doxorubicin, Aging, Cardiovascular diseases, Calcification

## Abstract

Senescence of vascular smooth muscle cells (VSMCs) contributes to aging as well as age-related diseases of the cardiovascular system. Senescent VSMCs have been shown to be present in atherosclerotic plaques. Both replicative (RS) and stress-induced premature senescence (SIPS) accompany cardiovascular diseases. We aimed to establish the signature of RS and SIPS of VSMCs, induced by a common anticancer drug, doxorubicin, and to discover the so far undisclosed features of senescent cells that are potentially harmful to the organism. Most of the senescence hallmarks were common for both RS and SIPS; however, some differences were observed. 32 % of doxorubicin-treated cells were arrested in the G2/M phase of the cell cycle, while 73 % of replicatively senescing cells were arrested in the G1 phase. Moreover, on the basis of alkaline phosphatase activity measurements, we show that a 7-day treatment with doxorubicin (dox), does not cause precocious cell calcification, which is a characteristic feature of RS. We did not observe calcification even though after 7 days of dox-treatment many other markers characteristic for senescent cells were present. It can suggest that dox-induced SIPS does not accelerate the mineralization of vessels. We consider that detailed characterization of the two types of cellular senescence can be useful in in vitro studies of potential anti-aging factors.

**Electronic supplementary material:**

The online version of this article (doi:10.1007/s10522-013-9477-9) contains supplementary material, which is available to authorized users.

## Introduction

It is believed that accumulation of senescent cells in tissues and organs causes multiple dysfunctions and can be the reason of age-related diseases (Chen et al. 2007). Increased number of senescent cells has been described in many species mainly in tissues which contain mitotically competent cells (Dimri et al. 1995; Jeyapalan et al. 2007).Their number in healthy old animals was estimated to be about 15 % (Wang et al. 2009), therefore, it seems that together with low grade inflammatory state they should be seriously considered as the reason of aging.

Cellular senescence is characterized by a series of morphological and physiological changes (Rattan 2012) including irreversible block of proliferation. Two types of cellular senescence could be distinguished: replicative (RS) and accelerated, stress-induced premature senescence (SIPS) (Toussaint et al. 2000b). The first one results from telomere shortening and/or loss of telomere function (von Zglinicki et al. 2003) and affects almost all proliferating somatic cell types in the organism (Sikora et al. 2011). The second one is generally independent of telomere erosion and is caused by oxidative, genotoxic and oncogenic stress. The common feature of both, SIPS and RS is DNA damage induced mostly by oxygen species (Passos and Von Zglinicki 2006). Some features of cellular senescence are universal for many types of cells and include: altered cell morphology, increased activity of senescence associated-β-galactosidase (SA-β-gal), accumulation of DNA damage foci and increased level of cyclin-dependent kinase inhibitors belonging to tumor suppressor pathways, namely p21—a transcriptional target of p53, and p16—an upstream activator of Rb (Rattan 2012; Ohtani et al. 2004). Phosphorylation of p53 followed by expression of p21 is a consequence of activation of the DNA damage response pathway (DDR). This signaling pathway can be induced by DNA double strand breaks in telomeric regions during SIPS or telomere shortening during RS (Campisi et al. 2001). Another feature of cellular senescence is the senescence associated secretory phenotype—SASP, which is characterized by the secretion of proinflammatory cytokines, which together with the process of inflammaging (Franceschi et al. 2000) can be responsible for low grade inflammatory state. Another feature of senescence concerns the epigenome (Calvanese et al. 2009). A global methylation of DNA decreases during senescence and, simultaneously, the promoters of some genes can be hypermethylated. Cellular senescence is also connected with chromosomal instability. Aneuploidy or polyploidy are observed frequently in the cells of aging organisms and are correlated with decreased potential of DNA repair machinery (Sedelnikova et al. 2004).

Cellular senescence plays an important role in cardiovascular diseases (CVD), which remain the leading cause of death, and aging is one of the main risk factors for CVD development. Both epidemiological and autopsy studies suggest a close link between aging and clinical manifestation of atherosclerosis (Gorenne et al. 2006) and the reason is increased production of free radicals (Higashi et al. 2012). It is well documented that senescent vascular smooth muscle cells (VSMCs), and endothelial cells (ECs), together with low grade inflammation, are involved in CVD (Gorenne et al. 2006; Veronica and Esther 2012).

The SA-ß-gal positive VSMCs, as well as cells with DNA double strand breaks, were found in atherosclerotic plaques supporting the notion that vascular cell senescence plays a critical role in the age-related vascular disorder (Mercer et al. 2007). Numerous studies on CVD such as atherosclerosis or hypertension have evidenced the senescence of VSMCs and ECs as one of the contributing factors. Some data suggest that activation of DDR is a critical regulator of VSMC senescence (Matthews et al. 2006). DNA damage is augmented in cells isolated from atherosclerotic plaques as well as in the circulating cells of patients with atherosclerosis (Mercer et al. 2007).

There are also some cellular features characteristic only for senescent vascular cells (cell-type exclusive senescent phenotype—CESP), namely, an increased level of angiotensin II receptor and transdifferentiation of VSMCs to osteoblasts associated with the calcification process (Nakano-Kurimoto et al. 2009) or decrease in NOS activity in ECs (Minamino et al. 2002).

In this study we compared the signatures of RS and SIPS of human VSMCs. To our knowledge there is no such complex study involving analysis of both types of senescence performed on the same cell culture of VSMCs. SIPS was induced by doxorubicin. Doxorubicin has been used as a common anticancer drug for almost 40 years (Sun et al. 2013 and references herein). Several mechanisms of doxorubicin-induced DNA damage are known, which include: intercalation into DNA (leading to inhibited synthesis of macromolecules), generation of free radicals (leading to DNA damage and/or lipid peroxidation), DNA binding and alkylation, DNA cross-linking, interference with DNA unwinding or DNA strand separation, altered helicase activity and initiation of DNA damage via inhibition of topoisomerase II (Gewirtz 1999). Accordingly, we used doxorubicin as a factor inducing DNA damage, which can lead to DNA damage-induced senescence. There are some data showing that doxorubicin can induce premature senescence in cardiomyocytes (Maejima et al. 2008) and cardiac progenitor cells (Piegari et al. 2013), therefore we suggest that a similar side effect can be observed in VSMCs. We analyzed both the commonly used and unique markers of senescence. We found some differences between expression of those markers in RS and dox-induced SIPS in this type of cells.

## Materials and methods

The expanded version of this section can be found in the online-only Supplement.

Here briefly:

Human VMSCs were purchased from Lonza and ATCC (normal diploid cells derived from young males, at least from three different donors) and were cultured in SmBM medium (Lonza) or Vascular Cell Basal Medium (ATCC) supplemented as defined by the manufacturer. The cells were passaged every 3–4 days to analyze RS or were seeded 24 h before treatment (dox-induced SIPS analysis) at a density of 3–3.5 × 10^3^ cells/cm^2^.

*DNA content and cellular granularity* analysis were performed using FACSCalibur and CellQuestPro software as described by Korwek et al. (2012).

For *DNA synthesis assay*, bromodeoxyuridine (BrdU, Sigma-Aldrich) was added to the medium (10 μM) for 24 h.

*Transcriptional rDNA activity* was assessed as the size of AgNOR silver deposits as described elsewhere (Howell and Black 1980). The analysis of interphase AgNORs of 100 AoSMCs was conducted by the morphometric method.

Detection of *Senescence Associated-β-galactosidase* (SA-β-gal) activity was performed according to Dimri et al. (1995).

Detection of *53BP1 foci* was performed by using primary anti-53BP1 polyclonal antibody (1:500) (Novus) and the anti-rabbit Alexa 488 secondary antibody (1:500) (Invitrogen). DNA was stained with DAPI.

The evaluation of *micronuclei generation* was performed using a BD™ Gentest Micronucleus Assay Kit using the standard protocol.

*Fluorescence in situ hybridization* (FISH). For *p53* tumor suppressor gene and *hTERC* gene visualization, p53 (17p13)/SE 17 probe and hTERC (3q26)/3q 11 probe (Kreatech) were used, respectively.

*Western blotting analysis.* Whole cell protein extracts were prepared according to Laemmli (1970). Nitrocellulose membranes were incubated with one of the primary antibodies: anti-ATM (1:1,000), anti-phospho-ATM Ser1981 and anty-H2AX (1:500) (Millipore); anti-p53, anti-p16 and anti-p21 (1:500) (Santa Cruz); anti-phospho-p53 Ser15 (1:500) (Cell Signaling); anti γ-H2AX Ser139 (1:1,000) (Abcam); anti-Poly(ADP-ribose)polymerase (PARP) (1:1,000) (Enzo); anti-actin (Sigma) (1:50,000) and secondary antibody conjugated with HRP (Dako) (1:2,000). The respective proteins were detected using the ECL system, according to the manufacturer’s instructions.

*Secretory phenotype* (IL-6, IL-8, VEGF) was analyzed by ELISA assay. Experiments were conducted according to the protocol provided by the manufacturer (R&D Systems).

*Alkaline phosphatase activity* (ALP) in whole cell lysates was determined using p-NPP. The results are presented in enzyme activity units defined as nmoles of p-NPP hydrolyzed per minute per milligram of total protein. Cells exposed to 50 μg/ml ascorbic acid (AA, Sigma) and 7.5 mM β-glycerophosphate (β-GP, Sigma) (AA/BGP) were used as a positive control (PC) of the calcification process (Shioi et al. 1995).

*Intracellular superoxide production* was assayed with 5 μM dihydroethidine and monitored in a fluorescence mode microplate reader and a fluorescence microscope equipped with a CCD camera.

*Global DNA methylation* was estimated as the 5-methyl-2′-deoxycytidine (5-mdC) level using High Performance Liquid Chromatography (HPLC). For global DNA methylation inhibition control a 24-h cell treatment with 5 μM 5-aza-2′-deoxycytidine (5-aza-dC) was used.

*DNMT1* (DNA methylotransferase) *quantification and activity assay* was performed using an EpiQuik™ DNMT1 Assay Kit and an EpiQuik™ DNA Methyltransferase Activity/Inhibition Assay Kit (Epigentek) using the standard protocol.

*The methylation status of the CpG islands* of the *p16*, *hTERT* and *RB1* genes was assessed by methylation-specific PCR (MS-PCR) according to the method of Kumari et al. (2009) with a minor modification.

*Telomere restriction fragment* (TRF) length (Southern blot analysis). DNA samples were extracted by the Genomic DNA purification kit (Gentra Puregene Blood Kit, QIAGEN) according to the manufacturer’s instructions. Mean TRF length was measured using the TeloTAGGG telomere length assay kit (Roche Molecular Biochemical) according to the manufacturer’s instructions.

*Telomere length* (Q-FISH with Human Chromosome Pan-Telomeric Probes). For telomere visualization, STAR^®^FISH Human Chromosome Pan-Telomeric Cy3-labeled Probes (Cambio) were used according to the manufacturer’s instructions. A standard Q-FISH analysis was used as described by Ourliac-Garnier and Londono-Vallejo (2011). Mean telomere area in interphase nuclei of VSMCs was measured with TFL-TELO (Telomere Measurements and Analysis). Telomere length was expressed as a mean telomere area per cell, which is an equivalent of the fluorescence area (number of pixels) occupied by a single spot.

*Statistical analysis* was performed using 2-tailed Student *t* test, ANOVA, Tukey’s a posteriori test or Mann–Whitney *U* test to examine differences between two groups. Data are presented as a mean ± SD. A value of *p* < 0.05 was considered statistically significant (*p < 0.05, **p < 0.01, ***p < 0.001). All graphs show the mean results from at least three independent experiments.

## Results

The analysis of RS was performed in vitro on early passages, 5–8 (young cells), middle passages, 10–13, late passages, 15–18 (senescent cells) and sometimes at passages 19–23 (extremely senescent cells). SIPS was induced by growing the cells at the passage 5–8 in the presence of doxorubicin (100 nM) and analysis was performed after 1, 3 and 7 days. The results for RS and dox-induced SIPS are presented alongside.

### Morphology, SA-β-gal activity and cell granularity

**Fig. 1:**
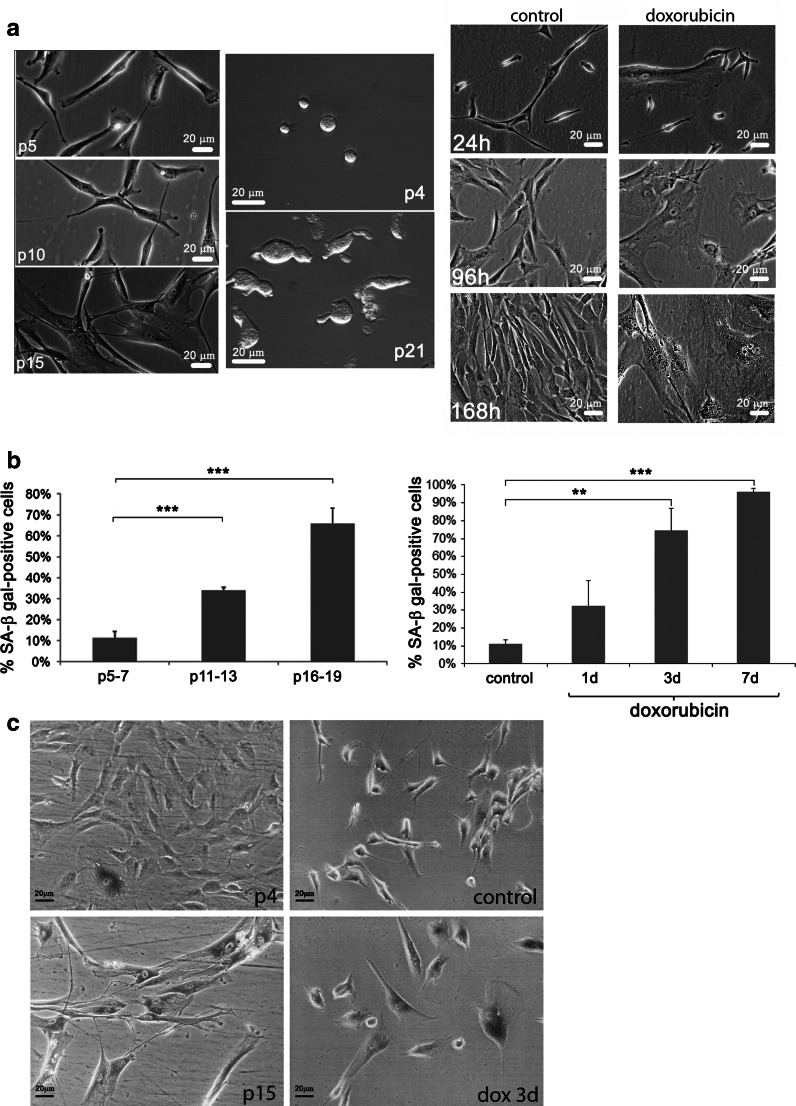
Cell morphology and SA-β-gal activity during young and senescent cells. **a** Morphology of cells in the culture (RS and dox-induced SIPS) and after detachment (RS). Representative pictures (*left*) of RS p5, p10, p15 cells growing at the culture dish and p4 and p21 detached cells. Cells treated with doxorubicin (*right*) after 24, 96 h (4 days) and 168 h (7 days) and, alongside, the corresponding pictures of control cells. Similar changes were observed during RS and dox-induced SIPS. The senescent cells were bigger and flatter than the young ones and after detachment their shape was irregular in contrast to round and small young cells. **b**, **c** SA-β-gal activity during RS (*left*) and dox-induced SIPS (*right*) expressed as a percentage of SA-β-gal-positive cells (**b**) and representative pictures of RS p4 and p15, and dox-induced SIPS (control cells and cells treated with doxorubicin during 3 days—dox 3 days) (**c**). The increased activity of SA-β-gal accompanied both RS and dox-induced SIPS. (*t* test)

The young cells at early passages were round and small while the senescent cells were big and their shape was irregular which was also visible after cell detachment from the bottom of the culture dish (Fig. 1a). Similar changes were observed during dox-induced SIPS.

The increased activity of SA-β-gal accompanied both RS and dox-induced SIPS. During RS the number of SA-β-gal positive cells increased with the passage number (p) and accounted for about 11, 34, 66 % of cells in p5–7, 11–13, 16–19, respectively (Fig. 1b). After doxorubicin treatment the number of SA-β-gal-positive cells increased with time and amounted to about 32 and 74 % after 1 and 3 days, respectively. After 7 days almost 100 % of cells were SA-β-gal-positive (Fig. 1b). The representative pictures for both types of senescent cells are shown (Fig. 1c).

**Fig. 2:**
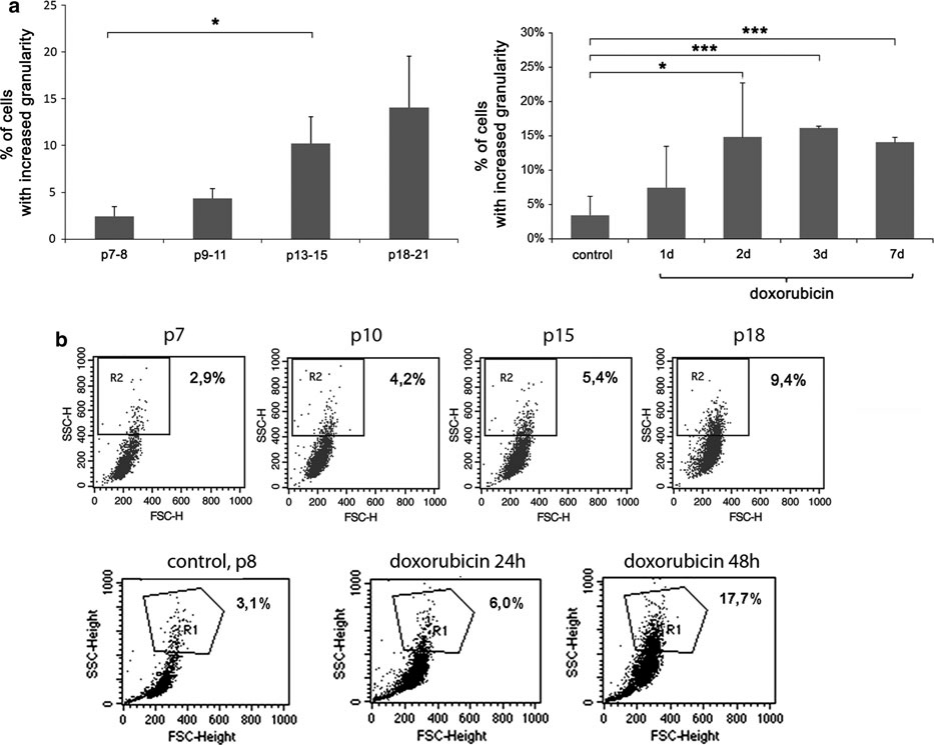
Changes of granularity of cells undergoing senescence. Granularity of cells during RS (*left*) and dox-induced SIPS (*right*) **a** expressed as a percentage of cells with increased granularity (*U* test and *t* test, respectively) and **b** representative dot blots obtained using flow cytometry, showing changes in cellular granularity during RS (*upper*), passages number 7, 10, 15, 18 and dox-induced SIPS (*lower*), with control cells (p8), and cells after 24 and 48 h of doxorubicin treatment, respectively. The region for analysis of cells with increased granularity was determined for passage 5 in the case of RS and for control cells in the case of doxorubicin treatment and was the same during all analyses. Cells present in the selected region reveal increased granularity in comparison to control. During RS and dox-induced SIPS an increased number of cells with enhanced granularity was observed

During RS and dox-induced SIPS an increased number of cells with enhanced granularity was observed (Fig. 2a). The percent of cells with increased granularity during RS was about 2, 4, 10, 14 % for p7–8, 9–11, 13–15, 18–21, respectively, and during dox-induced SIPS it was about 3 % in control, 8 % after 24 h and about 15 % at days 2–7 (Fig. 2a). The representative dot blots are shown in Fig. 2b.

### Cell proliferation and cell cycle

**Fig. 3:**
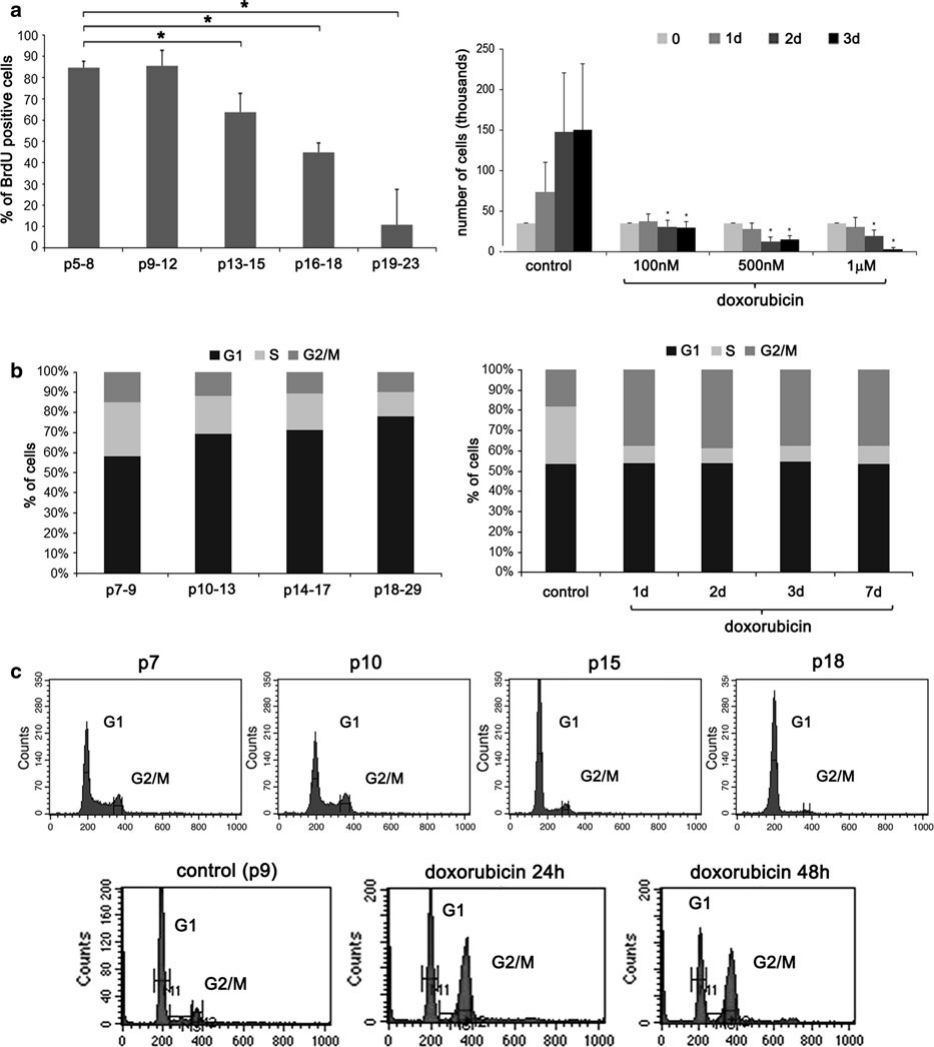
Estimation of proliferation rate and cell cycle during RS and dox-induced SIPS. **a** Proliferation rate during RS (*left*) and dox-induced SIPS (*right*), assessed as the percentage of BrdU positive cells (RS) (*U* test) or the number of cells in culture after doxorubicin treatment (dox-induced SIPS) (*t* test). During both RS and dox-induced SIPS cells stopped to proliferate. For the senescence induction different concentrations of doxorubicin were used. The 100 nM concentration was chosen as it did not induce massive cell death but effectively inhibited cell proliferation. **b** and **c** Cell cycle analysis during RS (*left* and *upper panel*) and dox-induced SIPS (*right* and *bottom* *panel*). Graphs showing % of cells in a particular phase of the cell cycle (**b**) and representative histograms showing preferential accumulation of cells in the G1 (RS) or the G2/M (dox-induced SIPS) phase of the cell cycle (**c**)

The PD (population doubling) and cPD (cumulative population doubling) were analyzed during RS. VSMCs divided about 35 times which corresponded to 18–20 passages. To further analyze the proliferation rate during RS the BrdU incorporation was performed. The proliferation rate diminished gradually with the passage number. The number of BrdU-positive cells between p5 and 12 was similar (about 85 %). At p13–15 the number of cells replicating DNA decreased to about 64 %, at p16–18 to about 45 % and at p19–23 to about 10 % (Fig. 3a, left graph). Different concentrations of doxorubicin were studied to choose a dose which did not induce massive cell death but effectively inhibited cell proliferation. The concentration of 100 nM led to growth arrest very quickly. The number of cells treated with 100 nM doxorubicin seemed to be almost the same during the whole time of culture and almost equal to the number of seeded cells. The higher doses, 500 nM and 1 μM, induced cell death which could be observed after 24 and 48 h. Therefore all experiments were performed using 100 nM doxorubicin (Fig. 3a, right graph).

Passage-dependent increase and doxorubicin-induced decrease in transcriptional rDNA activity, expressed as AgNOR areas, was observed. Extended description of the results is presented in the Supplement (Fig. 1).

The cell cycle analysis was performed. Percentage of cells in particular phases of the cell cycle for both RS and dox-induced SIPS is presented (Fig. 3b) and the representative histograms are shown (Fig. 3c). During RS we observed preferential accumulation of cells in the G1 phase of the cell cycle and after doxorubicin treatment cells preferentially accumulated in the G2/M phase of the cell cycle. During RS the percentage of cells in the G1 phase increased from about 56 % at p7–9 to 67 % at p10–17, and 73 % p18–21. At the same time the percentage of cells in the G2/M phase diminished from about 14 % at p7–9 to about 9 % at p18–21. Differences in the S phase of the cell cycle were also observed and the percentage of cells was about 26, 18, 17 and 12 % for cells between passages 7–9, 10–13, 14–17 and 18–21, respectively. In case of cells treated with doxorubicin the percentage of cells in the G1 phase of the cell cycle was about 51 % in control and 44–46 % after 1, 2, 3 and 7 days of treatment. The percentage of cells in the G2/M phase of the cell cycle increased from about 17 % (control) to 32 % and was the same later on. The differences in the number of cells in the S phase of the cell cycle were very spectacular. After doxorubicin treatment the percentage of cells in the S phase was about 7 % (from day 1 to day 7) in comparison to 27 % in control.

### Markers of DNA damage

It is believed that senescence of some types of cells is accompanied by chromatin instability and DNA damage. Therefore we checked if such events took place in the case of VSMCs senescence.

#### 53BP1 foci

**Fig. 4:**
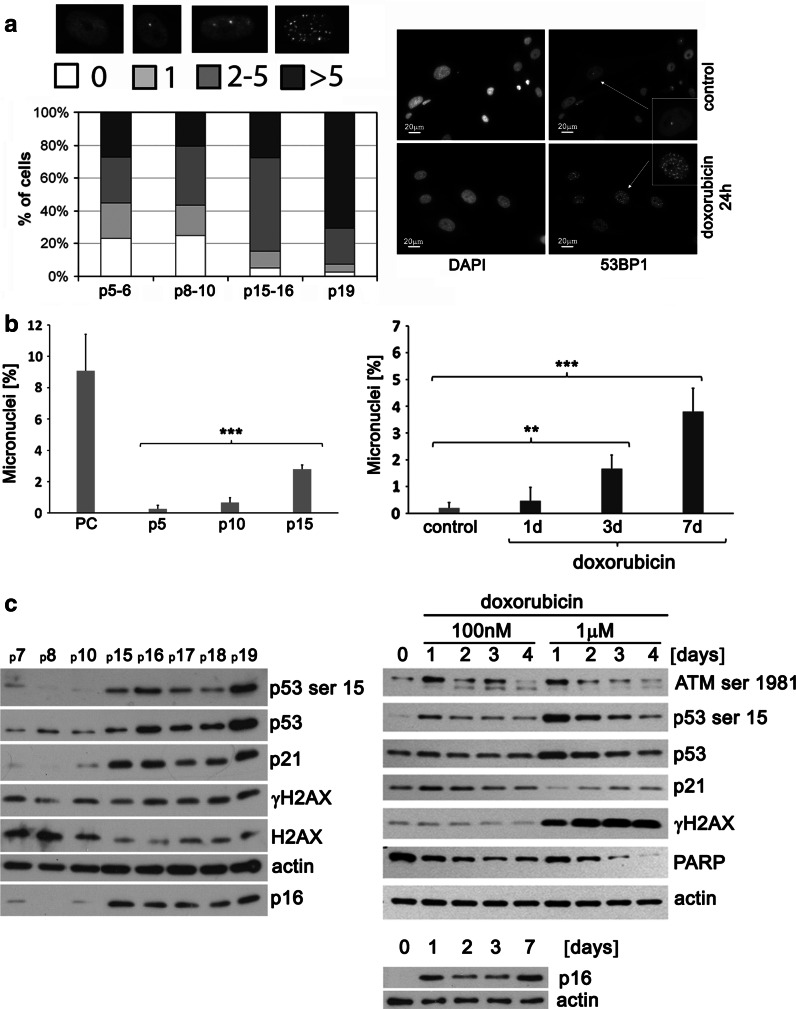
DNA damage during RS and dox-induced SIPS. **a** DNA damage expressed as the percentage of cells with DNA damage foci visualized by 53BP1 immunocytochemistry in RS (*left*), where the percentage of cells with 0, 1, 2–5 and more than 5 foci is shown, and representative pictures for dox-induced SIPS (*right*). During RS both the number of cells with damaged DNA and the number of DNA damage foci in particular cells increased; after 24 h of doxorubicin treatment in almost all cells more than 5 foci were found. **b** Micronuclei generation during RS (*left*) and dox-induced SIPS (*right*). During both RS and dox-induced SIPS a statistically significant time-dependent increase in micronuclei formation was observed. Cells treated with mitomycin C, which increased micronuclei generation, served as a PC. (ANOVA and Tukey’s a posteriori test). **c** Western blot analysis of DDR pathway and p16 level during RS (*left*) and dox-induced SIPS (*right*). An activation of the DDR pathway was observed during RS and dox-induced SIPS. An increased level of p53 and its phosphorylated form as well as p21 was observed. DDR activation during dox-induced SIPS was only transient. The level of p16 increased with the passage number and during dox-induced SIPS. Two concentrations of doxorubicin were analyzed, one used for dox-induced SIPS induction and a second one, 1 μM (added for 24 h), that led to cell death. In cells which mostly underwent senescence the level of both total and phosphorylated p53 was lower and the p21 expression increased already after 1 day but in cells treated with 1 μM doxorubicin such increase was observed later, only after 2 days. A very high level of γH2AX evidenced also the higher mortality of cells treated with 1 μM doxorubicin

The DNA double stand breaks (DSB) were visualized by immunocytochemical staining of 53BP1. Four categories of cells according to the number of foci were distinguished, namely, cells without DNA DSB, cells with only one focus, cells with two to five foci and cells with more than five foci. The DNA damage was observed in both RS and dox-induced SIPS. During RS both the number of cells with damaged DNA and the number of DNA damage foci in particular cells increased (Fig. 4a). Between p5 and 10 we found about 23 % of cells without DNA damage, about 5 % at p15–16 and less than 2 % at p19. The analysis showed that the number of cells with more than five foci was about 27 % at p5–6 but about 62 % at p19. The number of cells with only one focus was about 21, 18, 10 and 4 % for p5–6, 8–10, 15–16 and 19 respectively. The number of cells with 2–5 foci was about 28, 36, 57 and 19 % for p5–6, 8–10, 15–16 and 19, respectively. After 24 h of doxorubicin treatment in almost all cells more than five foci were found (Fig. 4a) and their number remained at the same level in the next days (not shown).

#### Micronuclei generation

Passage-dependent and doxorubicin-mediated micronuclei generation (a marker of genetic instability) was analyzed after blocking cytokinesis by cytochalasin B. Cells treated with mitomycin C served as a PC. Mitomycin C increased micronuclei generation by inhibition of DNA replication. We found a tenfold increase in micronuclei number in cells at p15 compared to cells at p5 (Fig. 4b). Doxorubicin induced a statistically significant time-dependent increase in micronuclei formation. After a 1-, 3- and 7-days treatment, we observed a 2-, 8- and 19-fold increase, respectively, in micronuclei formation compared to the control conditions. Despite a high fold increase in micronuclei generation, the percentage of cells forming micronuclei was quite low, e.g. 3.8 % after 7 days of doxorubicin treatment versus 0.2 % of control cells. Micronuclei formation was observed mainly in mononuclear cells (see micrographs Fig. 2 in the Supplement) both during RS and dox-induced SIPS and after mitomycin C treatment (PC). Micronuclei generation was slightly more augmented during dox-induced SIPS than RS.

#### Chromosomal aberrations

The passage-dependent and doxorubicin-induced changes in the chromosome number were analyzed. We observed some changes during RS and dox-induced SIPS concerning the *p53* gene and we did not observe any changes of the *hTERC* gene. The results are presented in the Supplement and Fig. 3.

### DDR pathway

Both RS and dox-induced SIPS are connected with DNA damage. DNA damage is followed by activation of the DDR and this signaling pathway was analyzed in both types of senescence. An increased level of p53 and its phosphorylated form as well as its target protein p21 was observed during RS (Fig. 4c). The level of a protein, not directly involved in DDR pathway but related to senescence, namely p16, increased with the passage number and during dox-induced SIPS. Transient activation of the DDR pathway was also observed during dox-induced SIPS (Fig. 4c). After treatment with 100 nM doxorubicin an increased level of phosphorylated ATM and p53 as well as p21 was observed already after 1 day and then gradually decreased. We analyzed two concentrations of doxorubicin; the one used for dox-induced SIPS induction and a second one, 1 μM (added for 24 h), tested earlier, that led to cell death (see Fig. 3a). During RS no substantial change in the level of γH2AX was observed. We observed only a subtle increase in the phosphorylated form accompanied by a simultaneous decrease in the total protein. During dox-induced SIPS (100 nM) we did not observe an increased level of γH2AX either. The elevation of the phosphorylated form of this histone was shown only after treatment with 1 μM doxorubicin. We compared the DDR pathway in cells undergoing senescence or apoptosis. Activation of the analyzed proteins varied in these two processes. Namely, in cells which mostly underwent senescence the level of both total and phosphorylated p53 was lower and the kinetics of p21 expression was different. In cells treated with 100 nM doxorubicin, p21 level increased already after 1 day. With 1 μM doxorubicin such increase was observed later, only after 2 days, while after 1 day the level was even lower than in the control. It can be explained by assuming that cells treated with a higher dose of doxorubicin were directed first to apoptosis and only those which survived, underwent senescence. Decreased level of PARP and a very high level of γH2AX evidenced also the higher mortality of cells treated with 1 μM doxorubicin. Such increase in γH2AX has been described for apoptotic cells (Korwek et al. 2012).

### Secretory phenotype

**Fig. 5:**
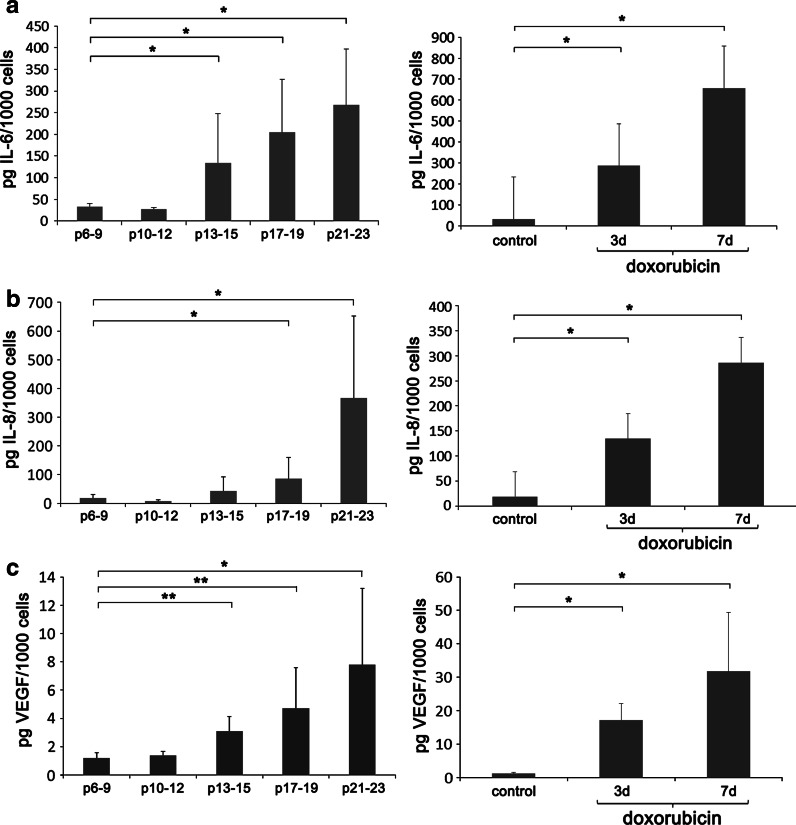
Secretory phenotype (SASP) during RS (*left*) and dox-induced SIPS (*right*). IL-6 (**a**), IL-8 (**b**) and VEGF (**c**) were analyzed. In both types of senescence an increased level of IL-6 and 8 and VEGF was observed and the production of all analyzed cytokines was higher during dox-induced SIPS than RS (*U* test)

In both types of senescence an increased level of IL-6 and 8 and VEGF was observed (Fig. 5a–c). During RS the production of: IL-6 increased from about 33 (p6–7) to 267 pg/1,000 cells (p21–23); IL-8 increased from about 19 (p6–7) to 366 (p21–23); VEGF increased from about 1 (p6–7) to 8 (p21–23) pg/1,000 cells. In the case of dox-induced SIPS the levels were compared to control (untreated cells p4–8). For IL-6 it was 33 (control) and 657 (7 days): for IL-8, 19 (control) and 286 (7 days): for VEGF 1,2 (control) and 32 (7 days) pg/1,000 cells. IL-1 was not detected in the culture medium. The production of all analyzed cytokines was higher during dox-induced SIPS than RS. It could be explained by the lower number of senescent cells undergoing RS than dox-induced SIPS (about 50 % of SA-β-gal positive cells at p16–19 and almost 100 % at day 7 after doxorubicin treatment).

### Calcification process

**Fig. 6:**
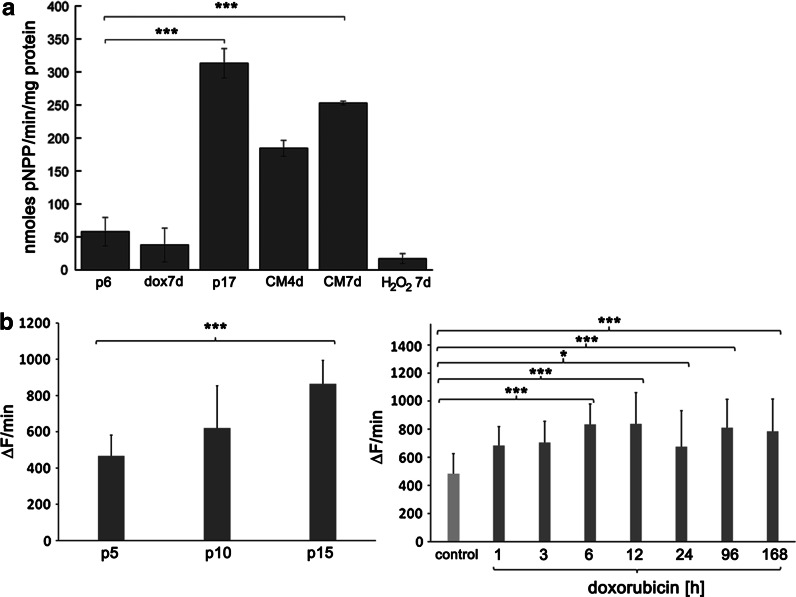
Analysis of ALP activity and superoxide production during RS and dox-induced SIPS. **a** ALP activity during RS and dox-induced SIPS is shown. ALP activity (expressed in nmoles of p-NPP released per minute (*U*) per milligram of protein). The results were normalized to the total protein concentration. dox7d—7 days treatment with doxorubicin, CM4d CM7d—4 or 7 days incubation in calcification medium, 100 μM H_2_O_2_ treatment for 7 days. The increased activity of ALP was observed only during RS. During SIPS induced by doxorubicin or H_2_O_2_ no increased activity of this enzyme was observed (Anova). **b** Superoxide production during RS (*left*) and dox-induced SIPS (*right*). ΔF/min—designates the increase in fluorescence per minute. Representative pictures are shown in Data Supplement. Intracellular superoxide production increased during both RS and dox-induced SIPS

The increased activity of ALP is the early marker of transdifferentiation to osteoblasts. Increased activity of this enzyme has been previously demonstrated in VSMCs in the presence of ascorbic acid (AA) and β-glycerophosphate (BGP). These two factors added together to the culture medium can induce diffuse calcification in a manner analogous to in vitro mineralization of osteoblasts (Shioi et al. 1995). Therefore cells treated with AA/BGP served as a PC (CM—calcification medium). The analysis of ALP revealed increased activity of this enzyme in cells undergoing RS. At p17 the activity was seven times higher than at p6 and was also higher than in the PC (Fig. 6a). However, in cells treated with doxorubicin no changes in ALP activity, in comparison to control cells (p6), were observed. It can suggest that the mineralization process does not accompany dox-induced SIPS. Therefore other known inductors of SIPS were studied, namely 100 μM H_2_O_2_ (Fig. 6a) and etoposide (not shown). The results were similar and no increase in the activity of ALP was observed.

### Superoxide production

Intracellular superoxide production was estimated by the rate of increase in ethidine fluorescence and the steady-state level of oxidation of dihydroethidine. In RS the superoxide production increased by about 85 % at p15 in comparison to p5 (Fig. 6b). Doxorubicin increased the superoxide production by about 40 and 73 % at 4 and 7 days of treatment, respectively, compared to the control. Similar results were recorded for the steady-state level of oxidation of dihydroethidine. A typical fluorescence micrographs showing passage-dependent and doxorubicin-induced superoxide steady-state level are presented at the Fig. 4 in the Supplement). We found that intracellular superoxide production increased during both RS and dox-induced SIPS.

### DNA methylation

Since senescence-associated decrease in total DNA methylation and hypermethylation of certain gene promoters was reported (Liu et al. 2003), we analyzed the changes in global DNA methylation during RS and dox-induced SIPS as well as DNA methyltransferase 1 (DNMT1) level, total DNMT activity, and the methylation pattern of selected promoters.

#### Global DNA methylation

**Fig. 7:**
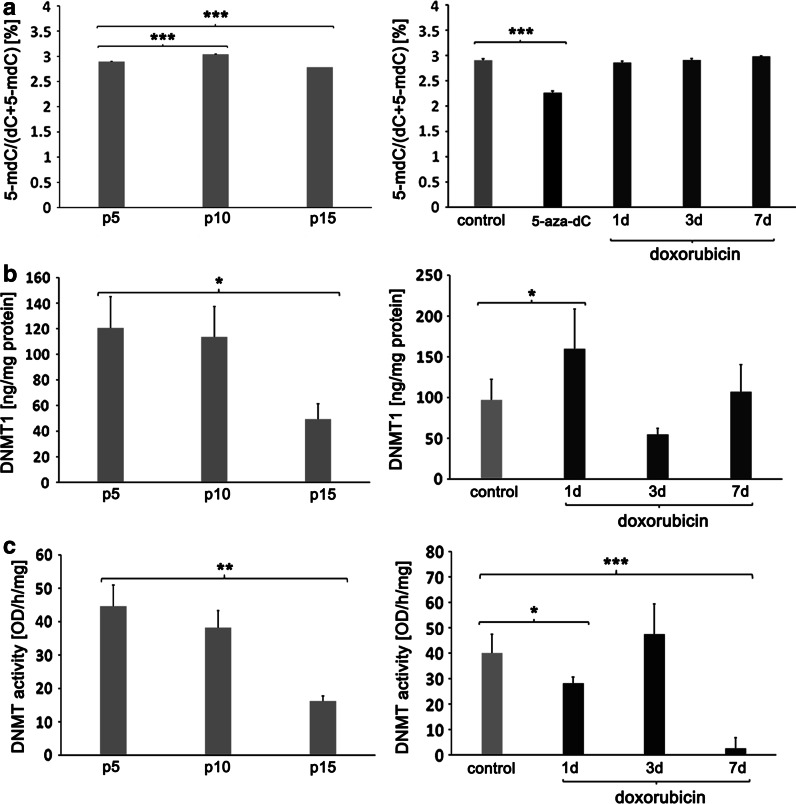
DNA methylation, DNMT1 level and DNMTs activity during RS and dox-induced SIPS. **a** DNA methylation. **b** DNMT1 level. **c** activity of DNMTs during RS (*left*) and dox-induced SIPS (*right*). DNA methylation of cells at p10 was higher than at p5, whilst DNA methylation of cells at p15 was lower than at p5. No statistically significant changes in DNA methylation in cells treated with doxorubicin was detected. Cells treated with a DNA methylation inhibitor 5-aza-2′-deoxycytidine (5-aza-dC) served as a negative control. We observed passage-dependent decrease in DNMT1 level and DNMT activity but during dox-induced SIPS some fluctuation in DNMT1 level and DNMT activity was observed (ANOVA and Tukey’s a posteriori test)

Cells treated with a well-known DNA methylation inhibitor 5-aza-2′-deoxycytidine (5-aza-dC) served as a negative control. DNA methylation of cells at p10 was about 3.5 % higher than that of p5 cells, whilst DNA methylation of p15 cells was about 4.3 % lower than that of p5 cells. We did not detect any statistically significant changes in DNA methylation of cells treated with doxorubicin. After 24 h treatment with 5 μM 5-aza-dC, we observed a 28 % inhibition in global DNA methylation (Fig. 7a).

#### DNMT1 level and DNMT activity

DNA methyltransferase 1 (DNMT1) is the primary enzyme responsible for maintenance of DNA methylation on genomic DNA (Liu et al. 2003). We observed passage-dependent decrease in DNMT1 level and DNMT activity (Fig. 7b, c). A 2.44-fold decrease of DNMT1 level and a 2.75-fold lower activity of DNMT were found in p15 cells compared to p5. During dox-induced SIPS changes in DNMT1 level and DNMT activity were more complicated and some fluctuation was observed. After 24 h of doxorubicin treatment a 65 % increase in DNMT1 level and simultaneously a 43 % decrease in DNMT activity were observed which may indicate a compensatory mechanism. After 4 days of treatment the level was lower and the activity was higher than in control. After 7 days, DNMT activity dramatically dropped (a 16-fold decrease). Nevertheless, DNMT1 content was not changed.

#### Analysis of methylation of selected promoters

We observed partial methylation of *hTERT* promoter during RS and dox-induced SIPS but we did not observe any methylation of *p16* and *RB1* promoters (data not shown). The results are presented in the Supplement. See also supplementary Fig. 5.

### Telomere length

**Fig. 8:**
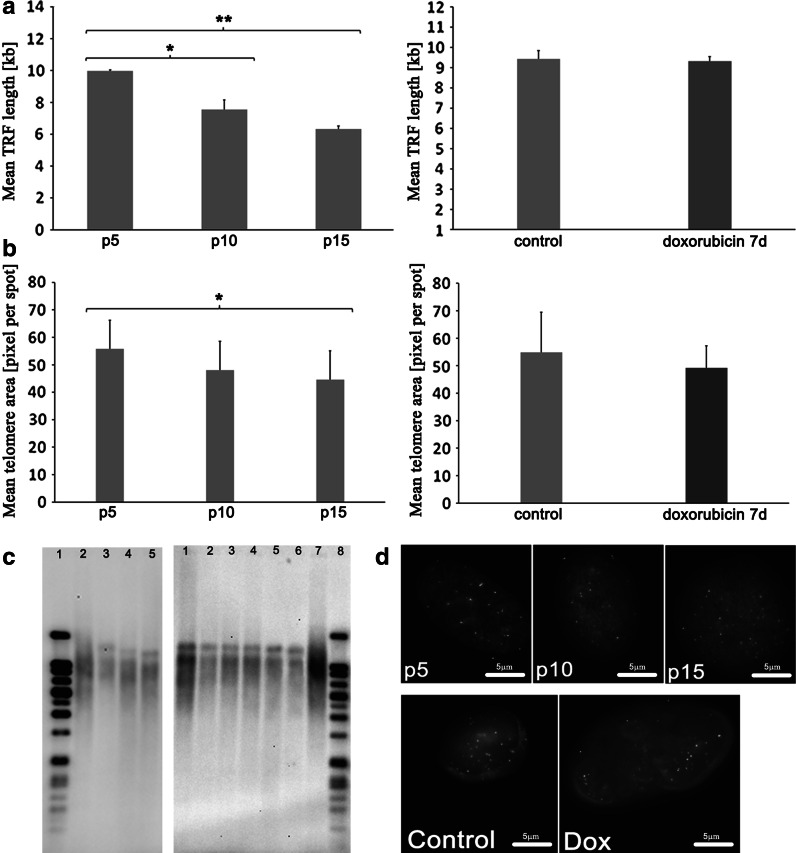
Telomere length during RS and dox-induced SIPS. **a** TRF length (kb) expressed as a mean telomere area per cell. **b** Means of telomere area (pixel per spot) for RS (*left*) and dox-induced SIPS (*right*) measured by Q-FISH with Human Chromosome Pan-Telomeric Probes, (ANOVA and Tukey’s a posteriori test). **c** Representative pictures from Southern blot analysis. Mean TRF length (kb) is shown in *brackets* (in the legend of the particular lanes). RS (*left*); *Lanes 1* DIG-Molecular Weight Marker, [0.8–21.2], *2* control DNA [7.6 ± 0.2], *3* p5 [9.97 ± 0.06], *4* p10 [7.55 ± 0.60], *5* p15 [6.33 ± 0.19]. dox-induced SIPS (*right*); *Lanes 1* p6 1 day, *2* doxorubicin 1 day, *3* p6 4 day, *4* doxorubicin 4 day, *5* p6 7 day, *6* doxorubicin 7 day, *7* control DNA [7.6 ± 0.2], *8* DIG-Molecular Weight Marker [0.8–21.2]. **d** Representative pictures of Q-FISH for passage-dependent and premature senescence induced by doxorubicin. A passage-dependent telomere length shortening was observed but no changes in the mean telomere length during dox-induced SIPS were recorded

Although telomere erosion is considered to be the reason of cell division limit, it has been also observed in SIPS (Toussaint et al. 2000b). We wanted to know if during dox-induced SIPS the telomere length could be altered. To this end we performed Southern blotting and Q-FISH. We observed a passage-dependent telomere length shortening (Fig. 8). With the TRF length test, we found a diminution in telomere length of about 32 and 58 % for cells from p10 and p15, respectively, compared to cells from p5 (Fig. 8a). The representative pictures are shown (Fig. 8c). Q-FISH revealed a mild, statistically significant, decrease in the mean telomere length during RS in cells from p15 compared to cells from p5 (a 25 % reduction) (Fig. 8b). Typical fluorescence micrographs are shown in the Fig. 8d. We did not record any changes in the mean telomere length during dox-induced SIPS.

## Discussion

In this paper we analyzed the signatures of RS and dox-induced SIPS of VSMCs in vitro. We checked a set of markers, some of which are commonly used for describing cellular senescence and are universal for most types of cells, but some were analyzed for the first time upon senescence of VSMCs.

A large body of literature concerns differences between senescence of cells isolated from atherosclerotic plaques in comparison with cells derived from parts of healthy vasculature (e.g. Matthews et al. 2006). It has been shown that VSMCs derived from human atherosclerotic plaques senesce faster than cells from normal vessels (Bennett et al. 1998). However, data concerning SIPS induced by chemotherapeutic drugs such as doxorubicin in cells building the vasculature are limited. It was reported that doxorubicin could initiate dox-induced SIPS in cardiomyocytes (Maejima et al. 2008; Spallarossa et al. 2009) and cardiac progenitor cells (Piegari et al. 2013). There is also one report concerning doxorubicin-induced senescence of VSMCs of the human umbilical artery, however, the experimental conditions were different than in our study—higher concentrations of doxorubicin, 0.25–1 μM were used, and all analyses were performed after 3 h of treatment (Hodjat et al. 2012). To our knowledge no such complex studies, concerning not only dox-induced SIPS but also RS of cells derived from the same donors and including so many analyses, were performed by other researchers.

We observed classical markers of cellular senescence in both RS and dox-induced SIPS. The common markers for both types of senescence were: SA-β-gal activity, DNA damage foci, DDR pathway activation, changed morphology, increased superoxide production, SASP, increased cell granularity and increased micronucleation. We observed no polyploidy in either type of senescence.

Despite some similarities between senescence signatures during RS and dox-induced SIPS we found some differences such as: the phase of cell cycle arrest, calcification, global DNA methylation and telomere length.

The first difference is the phase of the cell cycle, in which the cells preferentially accumulate, namely G1 during RS and G2/M during dox-induced SIPS. The explanation of this phenomenon could be that during RS the proliferation rate decreases gradually and cells do not enter mitosis because the telomere erosion induces the G1/S DNA checkpoint and therefore cells are mostly arrested in the G1 phase. During dox-induced SIPS cells which were in the S or G2 phase upon doxorubicin treatment did not complete the division and increased the population of cells in the G2/M phase. We cannot exclude that the activation of the tetraploid DNA checkpoint took place; we did not observe cells in mitosis (not shown). In the remaining cells, which were not in mitosis when doxorubicin was added, the DNA damage induced the G1/S checkpoint and they became arrested in the G1 phase. Therefore we observed cells in both G1 and G2/M. Since we observed an increased number of cells with 4N DNA content we cannot exclude the possibility that some cells were arrested not in the G2/M but in the 4NG1 phase of the cell cycle. We also observed very rare binucleated cells, which could suggest the 4NG1 phase of the cell cycle. Further analysis is necessary to distinguish the cell cycle phase of 4N DNA containing cells. Our result proved that accumulation of cells with a 4N DNA content can be used as a marker of premature senescence (Chatterjee et al. 2011).

The second difference between RS and dox-induced SIPS is the calcification. Vascular calcification is a clinically significant pathology in atherosclerosis and is associated with an increased level of extracellular mineral ions such as calcium and phosphate (Goodman et al. 2004). VSMCs play a key role in initiation and regulation of vascular calcification (Shanahan et al. 1999). The calcification process of VSMCs shares many similarities with that of bone formation (Demer and Tintut 2008). One of the proteins secreted by osteoblasts, which plays a central role in ECM calcification, is ALP, and the level of this enzyme was elevated during RS of VSMCs (Nakano-Kurimoto et al. 2009). It has been shown that both the expression and the activity of this protein increased during RS of VSMCs and that knockdown of ALP reduced calcification in senescent cells (Nakano-Kurimoto et al. 2009). We showed that the activity of ALP increased significantly during RS but did not increase during dox-induced SIPS. Similar results were obtained after treatment with other SIPS inducers such as etoposide (not shown) or hydrogen peroxide. It suggests that calcification does not accompany SIPS in this type of cell. However, we cannot exclude that after a longer time of post dox-treatment culture this characteristic feature will appear. Our conclusion comes from the observation that after 7 days of doxorubicin treatment other markers of senescence were already present while ALP activity, defined as an early marker of calcification, did not increase. Moreover, in the PC (cells treated with AA/BGP) the increased activity of ALP was visible already after 4 days. Thus, we demonstrated for the first time that doxorubicin induced senescence without calcification and that the extent of mineralization in the organism could be correlated with RS but not dox-induced SIPS.

The next difference concerns DNA methylation. Decreased global methylation of DNA is one of the molecular changes connected with aging (Bellizzi et al. 2012; Koch et al. 2011). We showed that global DNA methylation changed with the passage number but we did not observe any significant changes during dox-induced SIPS. Although global methylation of DNA decreases with age, the promoters of some genes can be hypermethylated. The age-related hypermethylation of the promoter regions of specific genes involved in cell cycle regulation, DNA repair, apoptosis, metabolism, cell signaling, plays a role in the age-related diseases (Richardson 2003). Methylation of DNA plays an important role also in CVD. We analyzed promoter methylation of selected age-related genes during RS and dox-induced SIPS. However, no significant changes in *p16* and *RB1* promoter methylation patterns were observed. Methylation regulates also the expression of the human telomerase reverse transcriptase gene (*hTERT*) (Kumari et al. 2009). We found for the first time that the *hTERT* gene promoter in VSMCs was partially methylated and there were no significant changes in *hTERT* methylation during RS and dox-induced SIPS.

We showed that the level of DNMT1 and the total DNMT activity decreased during RS but fluctuated during dox-induced SIPS. A decrease in DNMT1 level and total DNMT activity corresponds to global DNA hypomethylation during RS. Changes in DNMT1 level and total DNMT activity during dox-induced SIPS are rather a puzzle. Such phenomenon may be explained, at least in part, by changes in p21 level during 100 nM doxorubicin-induced SIPS. An inverse relationship between the expression level of DNMT1 and p21 in mammalian cell cultures was reported (Milutinovic et al. 2000). The process still awaits elucidation.

It was not surprising that telomere shortening took place only in RS. It was postulated previously that the mechanism of doxorubicin-induced SIPS depends on both functional p53 and telomere dysfunction rather than shortening, but such dysfunction can disrupt the telomere loop structure (Elmore et al. 2002). It has been demonstrated that cellular senescence can be the result of a prolonged arrest of cells in mitosis which in consequence may cause disorganization of telomere binding factors 2 (TRF2) and the telomere structure (Hayashi et al. 2012.). Doxorubicin is a regulator of the expression of TRF1 and TRF2 (Spallarossa et al. 2009). Telomere shortening is closely associated with increased severity of atherosclerosis (Matthews et al. 2006). It is widely accepted that telomere dysfunction plays a main role in the stress-induced senescence program and in apoptosis (Lechel et al. 2005). We can thus suppose that drug-induced premature senescence can support the progression of atherosclerosis although we did not observe telomere injury in doxorubicin treated cells.

All differences between the two types of senescence are presented in Table 1.

**Table 1:** The comparison of RS and dox-induced SIPS

	RS	dox-induced SIPS
Changes in morphology	+	+
Proliferation ratio	↓	↓
SA-β-gal activity	↑	↑
Number of DNA damage (53BP1) foci	↑	↑
Activation of DDR pathway	+	+
Secretory phenotype	↑	↑
**Calcification (ALP activity)**	**+**	**–**
**Cell cycle arrest**	**G1**	**G2/M**
Increased cell granularity	+	+
**Transcriptional rDNA activity**	**↑**	**↑↓**
Superoxide production	↑	↑
Micronucleus formation	↑ (low)	↑ (low)
DNMT1 level	↓	↑↓
DNMT activity	↓	↓↑
**Global DNA methylation**	**↓**	**–**
Chromosomal aberrations	↑	↑
**Telomere shortening**	**+**	**–**
hTERT methylation	–	–

The differences are bolded

It is difficult to determine if SIPS occurs in vivo because in the organism its symptoms are probably overlaid by RS. Presumably this is the reason of the lack of information whether in atherosclerotic plaques the cells undergo RS or SIPS. There are some data suggesting that SIPS could be a mechanism of accumulation of senescent-like cells in the organism (Toussaint et al. 2000a), and a marker such as DNA content can be useful to monitor and distinguish it.

To summarize, the markers of senescent VSMCs described by us can be helpful for future studies of potential anti-aging factors. This type of cells is a suitable model for such study because cellular senescence is known to give rise to some CVD. The knowledge concerning the senescence signature of these cells can be useful to define which parameters can be improved by the tested factors. We also showed that one of the most important processes responsible for aging of the vascular system—calcification, probably does not accompany premature senescence. This can suggest that during chemotherapy (with the use of, for example, doxorubicin) this process is not involved in vascular injury and mineralization. There are only few direct proofs that DNA damage induces senescence in VSMCs and in this manner contributes to atherosclerosis. There is, however, quite a lot of indirect information based on the finding that in atherosclerotic plaques a significant population of cells is senescent and bears DNA damage. Direct correlation between DNA damage and atherosclerosis has been shown in experimental animals e.g. rabbits in which oxidative DNA damage was found in plaques (Martinet et al. 2001). Our results show a direct link between DNA damage induced by doxorubicin and senescence. Considering doxorubicin as a common anticancer drug one cannot exclude that it can cause a side effect, that is, cellular senescence. However, the beneficial effects of curing cancer by using doxorubicin outweigh its possible harmful influence on the vasculature.

Is it possible to protect the organism from aging by removing senescent cells? It seems to be true. The first success in alleviating of the aging symptoms by intervention on a cellular level has been published. It has been shown that removal of senescent cells can protect against an aging phenotype and therefore lead to rejuvenating of the organism (Baker et al. 2011; Thannickal 2013 and references herein). Additionally it is the first direct evidence for the role of senescence in the organismal aging. Another proposed approach to alleviate the symptoms of aging is a protection form cellular senescence. We fully support the opinion which has been proposed by Bennett’s group (Matthews et al. 2006) that prevention of cellular senescence may be a novel therapeutic target in atherosclerosis.

## Electronic supplementary material

Below is the link to the electronic supplementary material.

Supplementary material 1 (TIFF 5038 kb)

Supplementary material 2 (TIFF 6373 kb)

Supplementary material 3 (TIFF 15071 kb)

Supplementary material 4 (TIFF 15069 kb)

Supplementary material 5 (TIFF 15068 kb)

Supplementary material 6 (DOC 110 kb)

## References

[CR1] Baker DJ, Wijshake T, Tchkonia T, LeBrasseur NK, Childs BG, van de Sluis B, Kirkland JL, van Deursen JM (2011). Clearance of p16Ink4a-positive senescent cells delays ageing-associated disorders. Nature.

[CR2] Bellizzi D, D’Aquila P, Montesanto A, Corsonello A, Mari V, Mazzei B, Lattanzio F, Passarino G (2012). Global DNA methylation in old subjects is correlated with frailty. Age (Dordr)..

[CR3] Bennett MR, Macdonald K, Chan SW, Boyle JJ, Weissberg PL (1998). Cooperative interactions between RB and p53 regulate cell proliferation, cell senescence, and apoptosis in human vascular smooth muscle cells from atherosclerotic plaques. Circ Res.

[CR4] Calvanese V, Lara E, Kahn A, Fraga MF (2009). The role of epigenetics in aging and age-related diseases. Ageing Res Rev.

[CR5] Campisi J, Kim SH, Lim CS, Rubio M (2001). Cellular senescence, cancer and aging: the telomere connection. Exp Gerontol.

[CR6] Chatterjee N, Kiran S, Ram BM, Islam N, Ramasarma T, Ramakrishna G (2011). Diperoxovanadate can substitute for H(2)O(2) at much lower concentration in inducing features of premature cellular senescence in mouse fibroblasts (NIH3T3). Mech Ageing Dev.

[CR7] Chen JH, Hales CN, Ozanne SE (2007). DNA damage, cellular senescence and organismal ageing: causal or correlative?. Nucleic Acids Res.

[CR8] Demer LL, Tintut Y (2008). Vascular calcification: pathobiology of a multifaceted disease. Circulation.

[CR9] Dimri GP, Lee X, Basile G, Acosta M, Scott G, Roskelley C, Medrano EE, Linskens M, Rubelj I, Pereira-Smith O (1995). A biomarker that identifies senescent human cells in culture and in aging skin in vivo. Proc Natl Acad Sci USA.

[CR10] Elmore LW, Rehder CW, Di X, McChesney PA, Jackson-Cook CK, Gewirtz DA, Holt SE (2002). Adriamycin-induced senescence in breast tumor cells involves functional p53 and telomere dysfunction. J Biol Chem.

[CR11] Franceschi C, Bonafè M, Valensin S, Olivieri F, De Luca M, Ottaviani E, De Benedictis G (2000). Inflamm-aging. An evolutionary perspective on immunosenescence. Ann N Y Acad Sci.

[CR12] Gewirtz DA (1999). A critical evaluation of the mechanisms of action proposed for the antitumor effects of the anthracycline antibiotics adriamycin and daunorubicin. Biochem Pharmacol.

[CR13] Goodman WG, London G, Amann K, Block GA, Giachelli C, Hruska KA, Ketteler M, Levin A, Massy Z, McCarron DA, Raggi P, Shanahan CM, Yorioka N (2004). Vascular calcification in chronic kidney disease. Am J Kidney Dis.

[CR14] Gorenne I, Kavurma M, Scott S, Bennett M (2006). Vascular smooth muscle cell senescence in atherosclerosis. Cardiovasc Res.

[CR15] Hayashi MT, Cesare AJ, Fitzpatrick JA, Lazzerini-Denchi E, Karlseder J (2012). A telomere-dependent DNA damage checkpoint induced by prolonged mitotic arrest. Nat Struct Mol Biol.

[CR16] Higashi Y, Sukhanov S, Anwar A, Shai SY, Delafontaine P (2012). Aging, atherosclerosis, and IGF-1. J Gerontol A.

[CR17] Hodjat M, Haller H, Dumler I, Kiyan Y (2012). Urokinase receptor mediates doxorubicin-induced vascular smooth muscle cell senescence via proteasomal degradation of TRF2. J Vasc Res.

[CR18] Howell WM, Black DA (1980). Controlled silver-staining of nucleolus organizer regions with a protective colloidal developer: a 1-step method. Experientia.

[CR19] Jeyapalan JC, Ferreira M, Sedivy JM, Herbig U (2007). Accumulation of senescent cells in mitotic tissue of aging primates. Mech Ageing Dev.

[CR20] Koch CM, Suschek CV, Lin Q, Bork S, Goergens M, Joussen S, Pallua N, Ho AD, Zenke M, Wagner W (2011). Specific age-associated DNA methylation changes in human dermal fibroblasts. PLoS ONE.

[CR21] Korwek Z, Sewastianik T, Bielak-Zmijewska A, Mosieniak G, Alster O, Moreno-Villaneuva M, Burkle A, Sikora E (2012). Inhibition of ATM blocks the etoposide-induced DNA damage response and apoptosis of resting human T cells. DNA Repair (Amst).

[CR22] Kumari A, Srinivasan R, Vasishta RK, Wig JD (2009). Positive regulation of human telomerase reverse transcriptase gene expression and telomerase activity by DNA methylation in pancreatic cancer. Ann Surg Oncol.

[CR23] Laemmli UK (1970). Cleavage of structural proteins during the assembly of the head of bacteriophage T4. Nature.

[CR24] Lechel A, Satyanarayana A, Ju Z, Plentz RR, Schaetzlein S, Rudolph C, Wilkens L, Wiemann SU, Saretzki G, Malek NP, Manns MP, Buer J, Rudolph KL (2005). The cellular level of telomere dysfunction determines induction of senescence or apoptosis in vivo. EMBO Rep.

[CR25] Liu L, Wylie RC, Andrews LG, Tollefsbol TO (2003). Aging, cancer and nutrition: the DNA methylation connection. Mech Ageing Dev.

[CR26] Maejima Y, Adachi S, Ito H, Hirao K, Isobe M (2008). Induction of premature senescence in cardiomyocytes by doxorubicin as a novel mechanism of myocardial damage. Aging Cell.

[CR27] Martinet W, Knaapen MW, De Meyer GR, Herman AG, Kockx MM (2001). Oxidative DNA damage and repair in experimental atherosclerosis are reversed by dietary lipid lowering. Circ Res.

[CR28] Matthews C, Gorenne I, Scott S, Figg N, Kirkpatrick P, Ritchie A, Goddard M, Bennett M (2006). Vascular smooth muscle cells undergo telomere-based senescence in human atherosclerosis: effects of telomerase and oxidative stress. Circ Res.

[CR29] Mercer J, Mahmoudi M, Bennett M (2007). DNA damage, p53, apoptosis and vascular disease. Mutat Res.

[CR30] Milutinovic S, Knox JD, Szyf M (2000). DNA methyltransferase inhibition induces the transcription of the tumor suppressor p21(WAF1/CIP1/sdi1). J Biol Chem.

[CR31] Minamino T, Miyauchi H, Yoshida T, Ishida Y, Yoshida H, Komuro I (2002). Endothelial cell senescence in human atherosclerosis: role of telomere in endothelial dysfunction. Circulation.

[CR32] Nakano-Kurimoto R, Ikeda K, Uraoka M, Nakagawa Y, Yutaka K, Koide M, Takahashi T, Matoba S, Yamada H, Okigaki M, Matsubara H (2009). Replicative senescence of vascular smooth muscle cells enhances the calcification through initiating the osteoblastic transition. Am J Physiol Heart Circ Physiol.

[CR33] Ohtani N, Yamakoshi K, Takahashi A, Hara E (2004). The p16INK4a-RB pathway: molecular link between cellular senescence and tumor suppression. J Med Invest.

[CR34] Ourliac-Garnier I, Londono-Vallejo A (2011). A telomere length analysis by quantitative fluorescent in situ hybridization (Q-FISH). Methods Mol Biol.

[CR35] Passos JF, Von Zglinicki T (2006). Oxygen free radicals in cell senescence: are they signal transducers?. Free Radic Res.

[CR36] Piegari E, De Angelis A, Cappetta D, Russo R, Esposito G, Costantino S, Graiani G, Frati C, Prezioso L, Berrino L, Urbanek K, Quaini F, Rossi F (2013). Doxorubicin induces senescence and impairs function of human cardiac progenitor cells. Basic Res Cardiol.

[CR37] Rattan SIS (2012). Cell senescence in vitro. Encyclopedia of life sciences (eLS).

[CR38] Richardson B (2003). Impact of aging on DNA methylation. Ageing Res Rev.

[CR39] Sedelnikova OA, Horikawa I, Zimonjic DB, Popescu NC, Bonner WM, Barrett JC (2004). Senescing human cells and ageing mice accumulate DNA lesions with unrepairable double-strand breaks. Nat Cell Biol.

[CR40] Shanahan CM, Cary NR, Salisbury JR, Proudfoot D, Weissberg PL, Edmonds ME (1999). Medial localization of mineralization-regulating proteins in association with Monckeberg’s sclerosis: evidence for smooth muscle cell-mediated vascular calcification. Circulation.

[CR41] Shioi A, Nishizawa Y, Jono S, Koyama H, Hosoi M, Morii H (1995). Beta-glycerophosphate accelerates calcification in cultured bovine vascular smooth muscle cells. Arterioscler Thromb Vasc Biol.

[CR42] Sikora E, Arendt T, Bennett M, Narita M (2011). Impact of cellular senescence signature on ageing research. Ageing Res Rev.

[CR43] Spallarossa P, Altieri P, Aloi C, Garibaldi S, Barisione C, Ghigliotti G, Fugazza G, Barsotti A, Brunelli C (2009). Doxorubicin induces senescence or apoptosis in rat neonatal cardiomyocytes by regulating the expression levels of the telomere binding factors 1 and 2. Am J Physiol Heart Circ Physiol.

[CR44] Sun J, Sun G, Meng X, Wang H, Luo Y, Qin M, Ma B, Wang M, Cai D, Guo P, Sun X (2013). Isorhamnetin protects against doxorubicin-induced cardiotoxicity in vivo and in vitro. PLoS One.

[CR45] Thannickal VJ (2013). Mechanistic links between aging and lung fibrosis. Biogerontology.

[CR46] Toussaint O, Dumont P, Dierick JF, Pascal T, Frippiat C, Chainiaux F, Magalhaes JP, Eliaers F, Remacle J (2000). Stress-induced premature senescence as alternative toxicological method for testing the long-term effects of molecules under development in the industry. Biogerontology.

[CR47] Toussaint O, Medrano EE, von Zglinicki T (2000). Cellular and molecular mechanisms of stress-induced premature senescence (SIPS) of human diploid fibroblasts and melanocytes. Exp Gerontol.

[CR48] Veronica G, Esther RR (2012). Aging, metabolic syndrome and the heart. Aging Dis.

[CR49] von Zglinicki T, Petrie J, Kirkwood TB (2003). Telomere-driven replicative senescence is a stress response. Nat Biotechnol.

[CR50] Wang C, Jurk D, Maddick M, Nelson G, Martin-Ruiz C, von Zglinicki T (2009). DNA damage response and cellular senescence in tissues of aging mice. Aging Cell.

