# Supplementary figures and images for: A comparison of replicative senescence and doxorubicin-induced premature senescence of vascular smooth muscle cells isolated from human aorta
Source: Biogerontology. 2013 Nov 16;15(1):47–64. doi: 10.1007/s10522-013-9477-9 (PMC3905196; doi:10.1007/s10522-013-9477-9)

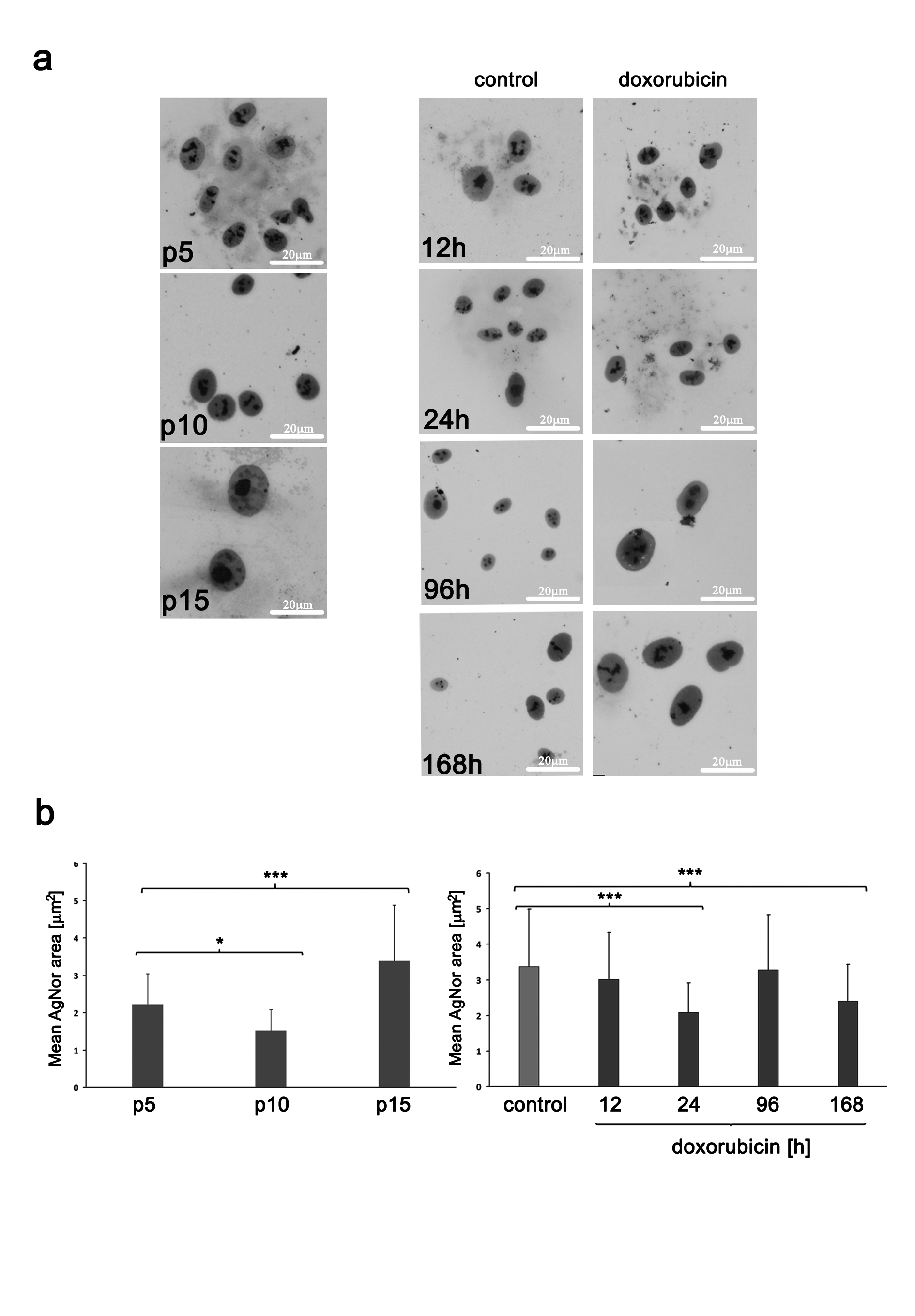

Supplement: Supplementary file 1 — Supplementary material 1 (TIFF 5038 kb) [file 10522_2013_9477_MOESM1_ESM.tif]

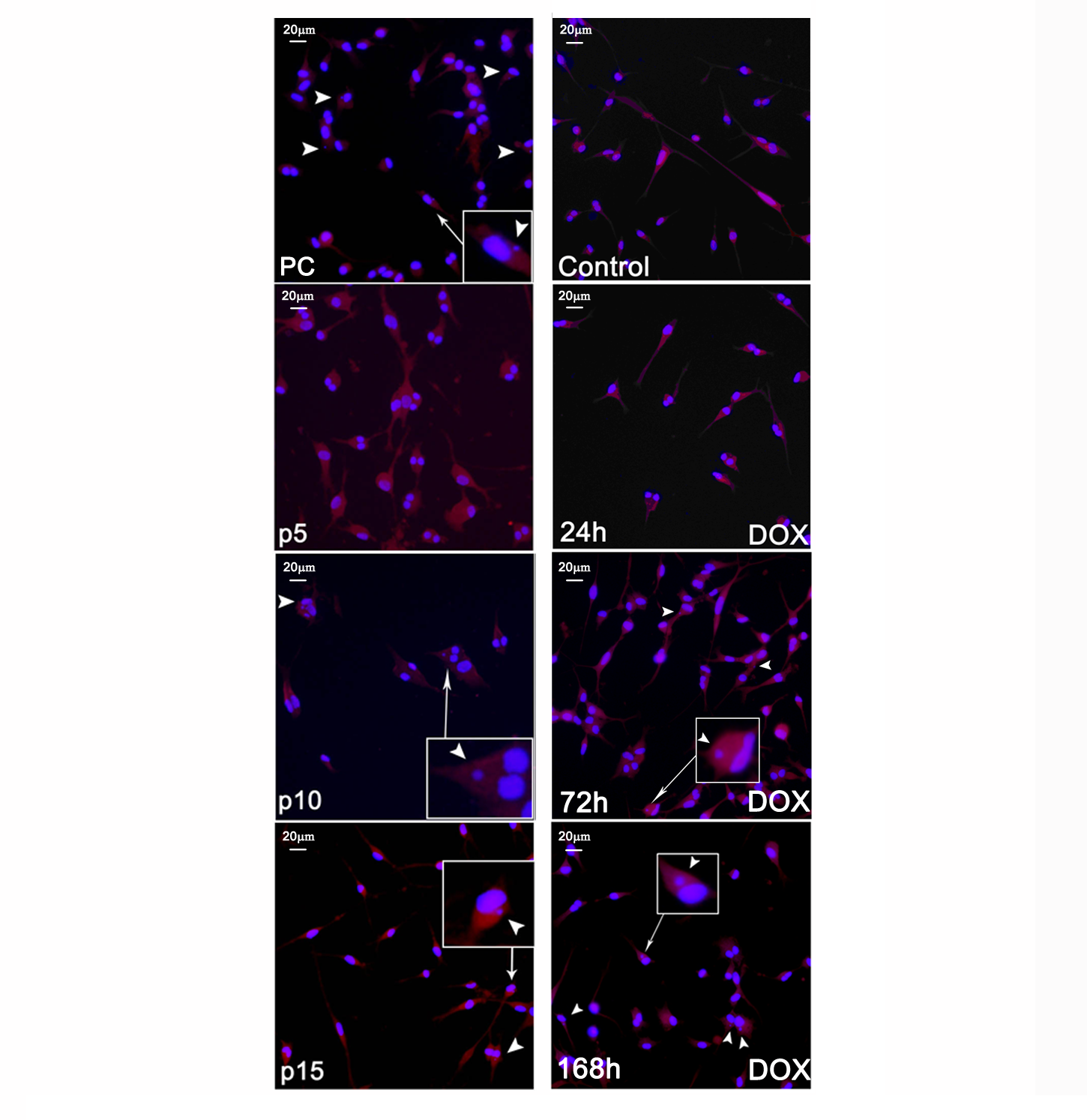

Supplement: Supplementary file 2 — Supplementary material 2 (TIFF 6373 kb) [file 10522_2013_9477_MOESM2_ESM.tif]

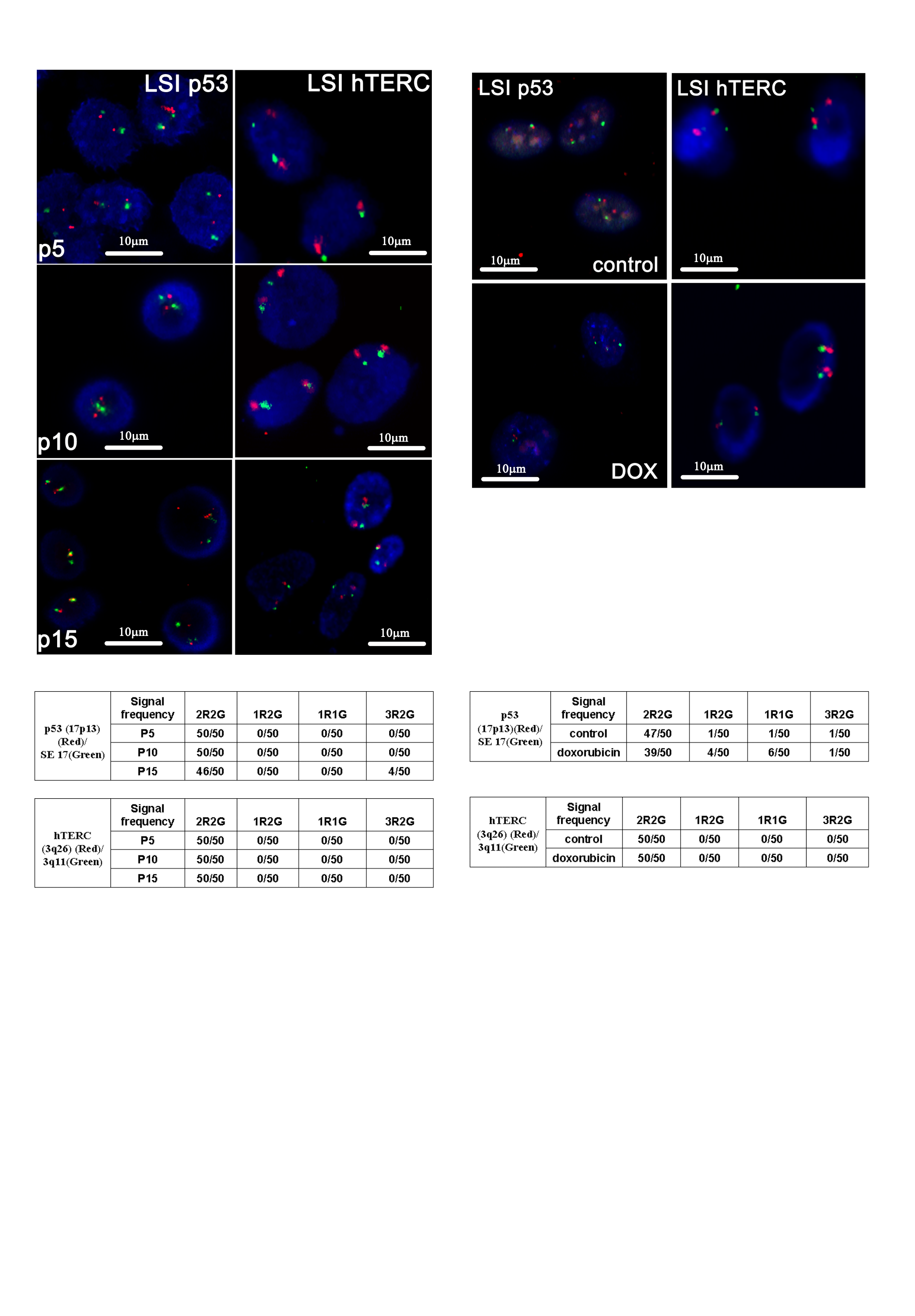

Supplement: Supplementary file 3 — Supplementary material 3 (TIFF 15071 kb) [file 10522_2013_9477_MOESM3_ESM.tif]

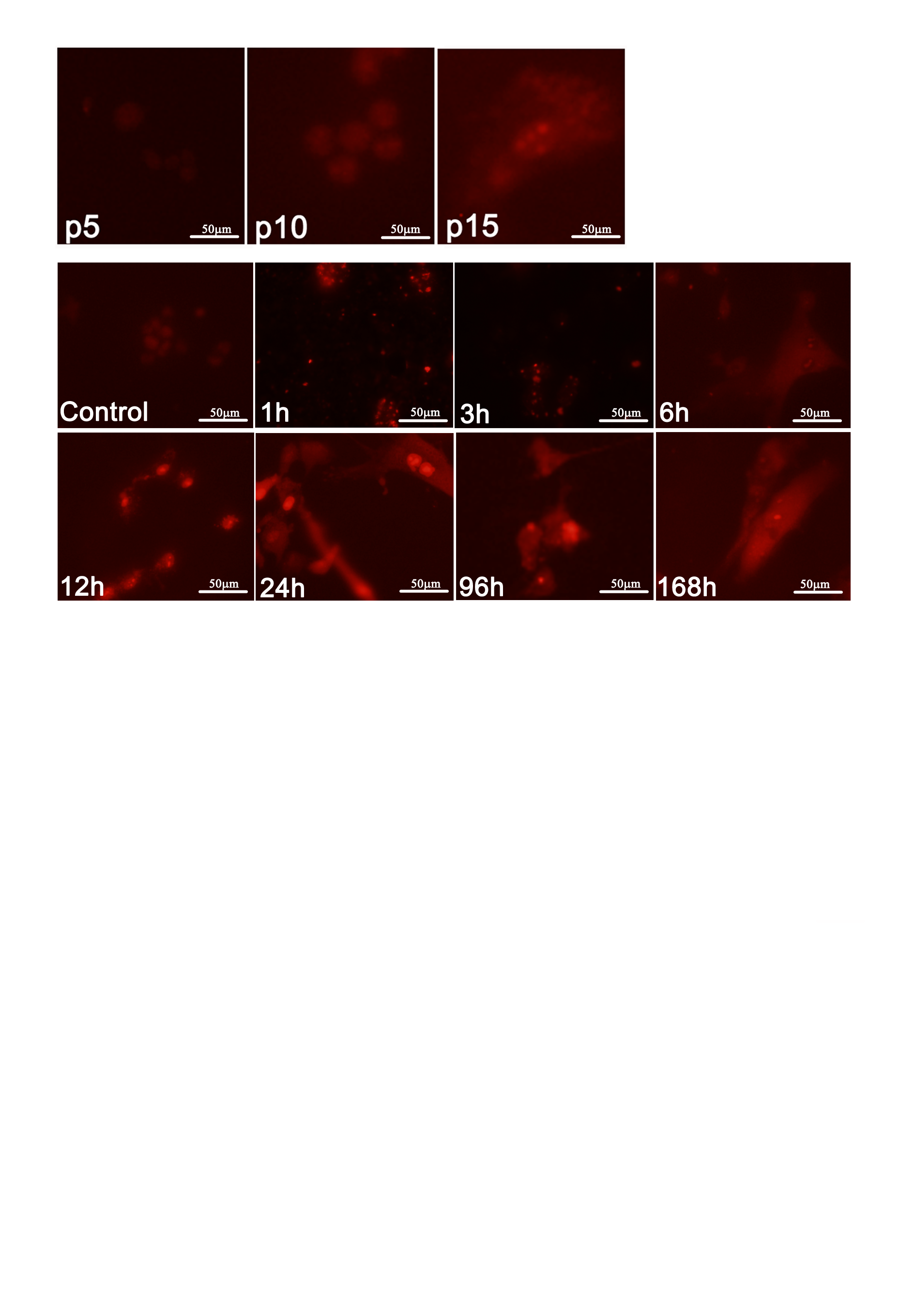

Supplement: Supplementary file 4 — Supplementary material 4 (TIFF 15069 kb) [file 10522_2013_9477_MOESM4_ESM.tif]

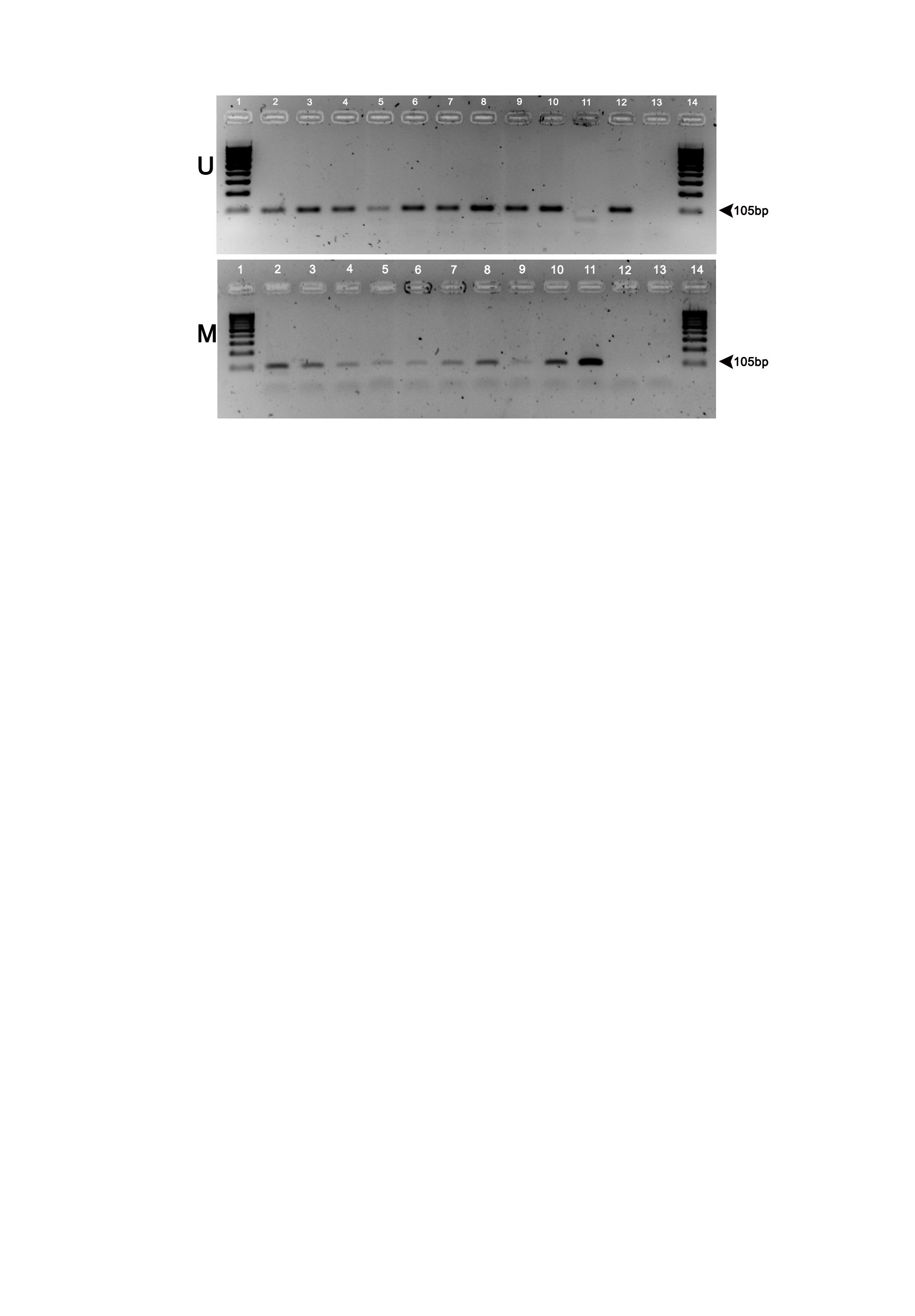

Supplement: Supplementary file 5 — Supplementary material 5 (TIFF 15068 kb) [file 10522_2013_9477_MOESM5_ESM.tif]
