# Supplementary material for: A comparison of replicative senescence and doxorubicin-induced premature senescence of vascular smooth muscle cells isolated from human aorta
Source: Biogerontology. 2013 Nov 16;15(1):47–64. doi: 10.1007/s10522-013-9477-9 (PMC3905196; doi:10.1007/s10522-013-9477-9)
Supplement: Supplementary file 6 — Supplementary material 6 (DOC 110 kb) [file 10522_2013_9477_MOESM6_ESM.doc]

###### Materials and methods

###### Reagents

SmBM medium with all supplements was purchased from Lonza (Lonza, Switzerland), Vascular Cell Basal Medium with all supplements was purchased from ATCC (LGC Standards, Poland), etoposide (E13830), dimethyl sulfoxide (DMSO) (D4540), BSA and anti β-actin (AC-15, mouse mAb, A-5541) were purchased from Sigma-Aldrich (Poznan, Poland). Anti γ-H2AX (Ser139, ab18311) (Abcam, Cambridge, UK), anti-H2AX (07-627 rabbit), anti-ATM (rabbit pAb, 07-1286) and anti-phospho-ATM (Ser 1981, mouse mAb, 05-740) were from Millipore (Warsaw, Poland); anti p53 (DO-1, mouse mAb, sc-126), anti p21 (C-19, rabbit pAb, sc-397) and anti-p16 (mouse, sc 1661) were from Santa Cruz Biotechnology (Santa Cruz, USA); anti phospho-p53 (Ser 15, Cell signaling, rabbit pAb, 9284), anti-Poly(ADP-ribose)polymerase (PARP) (mouse mAb,SA-249) from Enzo Life Sciences (Exeter, UK), horseradish peroxidase–conjugated secondary antibodies (P0448 and P0447) were from Dako (Gdynia, Poland); 53BP1, (pAB NB 100-304 rabbit) (Novus Biological, Cambridge UK), Alexa Fluor 488 (goat anti-mouse, A11001) from Invitrogen (Eugene, USA), ECL Western Blotting Detection Reagents (RPN2209) were from GE Healthcare (Buckinghamshire, UK); DRAQ5 (1,5-bis{[2-(di-methylamino) ethyl]amino}-4, 8-dihydroxyanthracene-9,10-dione) (BOS-889-001) was from Biostatus Limited (Leicestershire, UK); DAPI, Vectashield Mounting Medium For Fluorescence with DAPI (Vector Laboratories, Inc. Burlingeme, CA 94010).

# Collection and culture of vascular smooth muscle cells

Human VMSCs were purchased from Lonza and were cultured in SmBM medium or from ATCC and cultured in Vascular Cell Basal Medium supplemented as defined by the manufacturer. All cells were kept in humidified atmosphere (37°C and 5% CO_2_ in the air). The maximum lifespan was more than 3 months, cells divided about 30 times (cPD) and cells stooped to proliferate between 16 and 19 passage. PD were calculated by using the formula:

PD = [log(Nh)-log(Ni)]/log(2)

Ni – inoculum number (mln.ml)

Nh – cell harvest number (mln/ml)

The cells were passaged every 3-4 days to analyze replicative senescence or were seeded 24 hours before treatment (dox-induced SIPS analysis) at a density of 3-3,5x10^3^ cells/cm^2^ in all cases. Doxorubicin was dissolved in the medium. Cell morphology was analyzed in an inverted light microscope Nicon.

**Cell cycle and cell granularity analysis**

For DNA content and cellular granularity analysis as described by Korwek et.al. (Korwek et al. 2012). Cells were stained with PI solution and flow cytometry analysis of 10 000 cells was performed using FACSCalibur and the CellQuestPro software.

**Bromodeoxyuridine incorporation test**

For DNA synthesis assay, bromodeoxyuridine (BrdU, Sigma–Aldrich, Poznan, Poland) was added to the medium (10 µM) for 24 h. BrdU was detected by using primary antibody against BrdU (Becton Dickinson, DIAG-MED, Warsaw, Poland). In the case of doxorubicin treated cells the first analysis was performed after 48 h of treatment (BrdU added after 24 h of treatment). Cells were analyzed under a fluorescence microscope. Cells were counted and the % of BrdU positive cells is shown on graphs.

**Nucleolar organizer region (AgNOR) analysis**

Transcriptional rDNA activity, a parameter of cell proliferation, reflecting the physiological state of the cell, was assessed as the size of AgNOR silver deposits. The analysis was performed by silver staining of nucleolar organizer regions (AgNORs) according to Howell and Black (Howell and Black 1980). The analysis of interphase AgNORs of 100 VSMCs was conducted with the morphometric method according to Derenzini and Trere and following the guidelines of the Committee on AgNOR Quantification, (Derenzini and Trere 1991; Aubele et al. 1994) using the Olympus CellF software. Interphase NORs activity was expressed as a mean area of silver deposits which reflects cellular rDNA transcriptional activity and is related to cell-proliferation activity (Schmiady et *a*l. 1979; Derenzi*n*i et al. 2000).

**Estimation of Senescence Associated-β-galactosidase activity**

Detection of Senescence Associated-β-galactosidase (SA-β-gal) activity was performed according to Dimri et al. (Dimri et al. 1995). Cells were analyzed in a light microscope. Then cells were counted and the % of SA-β-gal-positive cells is shown.

**Immunocytochemistry**

For detection of 53BP1 foci cells were fixed with 70% ethanol and next incubated with primary anti-53BP1 antibody diluted 1:500 and with Alexa 488 secondary antibody, 1:500. DNA was stained with DAPI. 53BP1 foci were visualized under a fluorescence microscope.

**Cytokinesis-block micronucleus (CBMN) assay**

The evaluation of micronuclei generation was performed using a BD™ Gentest Micronucleus Assay Kit (DIAG-MED, Warsaw, Poland) following the standard protocol provide by the manufacturer. Micronuclei [%] were scored according to the criteria recommended in Kirsch-Volders et al. (Kirsch-Volders et al. 2003). At least 500 cells were analyzed in each of the three independent cultures used. To ensure the reproducibility of the assay, a positive control was always applied (24-h treatment with 100 ng/ml mitomycin C).

**Fluorescence *in situ* hybridization (FISH): *p53* and *hTERC* gene visualization**

For *p53* tumor suppressor gene and *hTERC* gene visualization, p53 (17p13)/SE 17 probe and hTERC (3q26)/3q 11 probe (Kreatech, Syngen Biotech, Wrocław, Poland) were used, respectively.

**Western blotting analysis**

Whole cell protein extracts were prepared according to Laemmli (Laemmli 1970). Used primary antibodies: anti-ATM (1:1000), anti-phospho-ATM Ser1981 (1:500), anti-p53 (1:500), anti-phospho-p53 Ser15 (1:500), anti-p21 (1:500), anti γ-H2AX Ser139 (1:1000), anty-H2AX (1:500), anty-p16 (1:500), anti-Poly(ADP-ribose)polymerase (PARP) (1:1000), anty-actin (1:50000). The respective proteins were detected after incubation with one of the horseradish peroxidase-conjugated secondary antibodies (1:2000), using an ECL system, according to the manufacturer's instructions.

**Measurements of secreted factors**

Secretory phenotype was analyzed by ELISA in 1 ml of the culture medium collected from cell culture. Experiments were conducted according to the protocol provided by the manufacturer (R&D Systems, Biokom, Warsaw, Poland). Levels of cytokines (IL-6, IL-8, VEGF) in the samples were determined with the use of standard curves and normalized to cell number. Absorbance was measured at 450 nm in a Tecan Sunrise spectrophotometer (Tecan) and analyzed with the X-fluor 4 software.

**Alkaline phosphatase (ALP) activity assay**

Alkaline phosphatase activity in whole cell lysates was determined using freshly prepared 10 mM p-NPP (4-nitrophenyl phosphate disodium salt hexahydrate) in reaction buffer (25 mM glycine, 25 mM piperazine, pH 10.4). The reaction was started by addition of the reaction buffer to the cell lysates and incubation at 37°C. The absorbance was measured at 15 min intervals at 420 nm in a BioMate3 spectrophotometer (Thermo Electron Co.). The ALP activity was quantified using a molar absorption coefficient of 18.8 cm^-1^ mM^-1^. The protein content in particular fractions was previously measured by the Bradford assay (BioRad Laboratories). The results were normalized as enzyme units in nmoles of p-NPP hydrolyzed per minute per milligram of total protein. As a positive control for calcification 50 µg/ml ascorbic acid (AA, Sigma-Aldrich Poznan, Poland) and 7.5 mM β-glycerophosphate (β-GP, Sigma-Aldrich Poznan, Poland) were used. Calciﬁcation of VSMCs was induced according the method of Shioi et al. (Shioi et al. 2002) with minor modiﬁcations. Because the AA-BGP treatment did not inhibit cell proliferation, cells were passaged after 4 days.

**Measurement of superoxide level**

Intracellular superoxide production was assayed with 5 μM dihydroethidine and monitored in a fluorescence mode microplate reader Tecan Infinite® M200 and a fluorescence microscope equipped with a CCD camera.

**Global DNA methylation assay**

DNA methylation was estimated as the 5-methyl-2′-deoxycytidine (5-mdC) level using High Performance Liquid Chromatography (HPLC) as described elsewhere (Potocki et al. 2012). For global DNA methylation inhibition control, a 24 h cell treatment with 5 µM 5-aza-2′-deoxycytidine (5-aza-dC) was used.

**DNMT1 quantification and activity**

DNMT1 (DNA methylotransferase) quantification and activity assay was performed using an EpiQuik^TM^ DNMT1 Assay Kit and an EpiQuik^TM^ DNA Methyltransferase Activity/Inhibition Assay Kit (Epigentek, Gentaur, Gdańsk, Poland) according to the standard protocol provided by the manufacturer.

**Methylation Status of *hTERT*, *p16* and *Rb* Gene Promoters**

The methylation status of the CpG islands of the *p16, hTERT* and *RB1* genes was assessed by methylation-specific PCR (MS-PCR) according to the methods of Kumari et. al. and Berge et al. (Berge et al. 2010; Kumari et al. 2009) with a minor modification. Genomic DNA treated with Sss1 methyltransferase was used as a positive control for methylated DNA. Whole genome amplification (WGA) was used to obtain the positive control for unmethylated DNA.

**Telomere restriction fragment (TRF) length (Southern blot analysis)**

DNA samples were extracted from the cells by the Genomic DNA purification kit (Gentra Puregene Blood Kit, QIAGEN) according to the manufacturer's instructions. Mean TRF length was measured using the TeloTAGGG telomere length assay kit (Roche Molecular Biochemical, Indianapolis, USA) according to the manufacturer's instructions and method described elsewhere (Wnuk et al. 2013).

**Telomere length (Q-FISH with Human Chromosome Pan-Telomeric Probes)**

For telomere visualization, STAR®FISH Human Chromosome Pan-Telomeric Cy3-labeled Probes (Cambio, UK) were used according to the manufacturer's instructions. A standard Q-FISH analysis was used as it was described by Ourliac-Garnier I et al. (Ourliac-Garnier and Londono-Vallejo 2011). Mean telomere area in interphase nuclei of VSMCs was measured with the TFL-TELO (Telomere Measurements and Analysis) Telomere length was expressed as a mean telomere area per cell, which is an equivalent of the fluorescence area (number of pixels) occupied by a single spot).

**Results**

**Transcriptional rDNA activity**

The AgNORs technique, reflecting the transcriptional rDNA activity, is mainly used to identify nucleoli in interphase nuclei as a parameter of cell proliferation rate and a significant marker in tumor pathology. On the other hand, since stress may inhibit cellular rRNA synthesis, its application as a marker of stress induction can be considered. Analysis of interphase AgNORs of 100 VSMCs was conducted with the morphometric method according to the guidelines of the Committee on AgNOR Quantification. Passage-dependent increase and doxorubicin-induced decrease in transcriptional rDNA activity, expressed as AgNOR areas, were observed (Sup.Fig. 1). We observed a 1.7-fold increase in the mean AgNOR area of cells at passage 15 compared to passage 5 (*p*<0.001). Majority of cells at passage 15 exhibited one enlarged nucleolus. Nevertheless, cells from passage 10 were characterized by decreased AgNOR expression compared to cells from passage 5 which may indicate a diminution in the cell proliferative rate. Doxorubicin-associated changes in the nucleolus size are shown. A 24 h and 7 day doxorubicin treatment caused about 60% and 40% decrease in the nucleolus area compared to the control conditions (*p*<0.001), respectively. After short doxorubicin treatment (12 h), we found a mild decrease (12%) in the mean AgNOR area compared to the control conditions, but the effect was statistically insignificant. After a 7-day doxorubicin-treatment some cells exhibited also enlarged, round-shaped nucleoli even though the mean nucleolus size of these cells was lower than of control cells.

**Chromosomal aberration analysis**

We analyzed the passage-dependent and doxorubicin-induced changes in the chromosome number. Two chromosomes (chr.17 and chr. 3) carrying *p53* and *hTERC* genes, respectively, were selected as markers of chromosomal aberrations (chromosome loss and gain). The two selected genes are crucial in cellular senescence and organismal aging. We observed passage-dependent amplification of *p53* gene, whilst doxorubicin was able to mediate various chromosomal aberration events such as deletion, amplification and aneuploidy (Sup.Fig. 3, see micrograph and table). Among all aberrations, aneuploidy events were the most manifested ones during dox-induced SIPS. We did not observe any changes involving *hTERC* gene. Taken together, chromosomal aberrations occurred more often during dox-induced SIPS than RS.

**Analysis of methylation of selected promoters**

To study promoter methylation pattern we selected genes suggested to be involved in cellular senescence such as *hTERT*, *p16* and *RB1*. We analyzed *hTERT* gene instead of *hTERC* gene since *hTERC* gene is not methylated in numerous healthy tissues (Liu et al.2003). We observed during both RS and dox-induced SIPS that *hTERT* promoter was partially methylated (Sup.Fig. 5) which may indicate that telomere shortening observed during RS is *hTERT* promoter methylation-independent. Contrary, two alleles of *p16* and *RB1* promoters were not methylated during RS or dox-induced SIPS (data not shown).

**References**

**Supplemental Figures**

**Legend**

**Sup.Fig. 1** Transcriptional rDNA activity, expressed as a number of AgNOR areas during RS (left) and dox-induced SIPS (right). (a) Representative micrographs, for at least three independent experiments, for RS and dox-induced SIPS. An increase in transcriptional rDNA activity during RS and decrease during dox-induced SIPS were observed (b). ANOVA and Tukey's *a posteriori* test

**Sup.Fig. 2** Micronuclei formation during RS (left) and dox-induced SIPS (right). Representative pictures for at least three independent experiments. To emphasize micronuclei formation, a magnification of the cell indicated by the arrow is provided. Arrowheads indicate cells with micronuclei. Left panel shows cells from passages 5, 10, 15 and PC indicate a positive control after mitomycin C treatment. Right panel shows cells after doxorubicin treatment, 24h, 72h (3 days), 168h (7 days). During both RS and dox-induced SIPS a statistically significant time-dependent increase in micronuclei formation was observed.

**Sup.Fig. 3** Passage-dependent and doxorubicin-mediated chromosomal aberrations. The representative micrographs show p53 (17p13)/ SE 17 probe and hTERC (3q26)/3q 11 probe hybridized to fixed VSMC nuclei during RS (left) and dox-induced SIPS (right). Tables show passage-dependent amplification of *p53* gene (left) and various aberrations detected with the p53 (17p13)/ SE 17 probe during dox-induced SIPS (right). Changes involving *hTERC* gene were recorded during RS (left) and dox-induced SIPS (right). Passage-dependent amplification of *p53* gene was observed. Doxorubicin was able to mediate various chromosomal aberration events such as deletion, amplification and aneuploidy. Among all aberrations, aneuploidy events were the most manifested ones during dox-induced SIPS. No changes involving *hTERC* gene were observed.

**Sup.Fig. 4** Superoxide production during RS and dox-induced SIPS. The micrographs show representative pictures for RS (upper) and dox-induced SIPS (lower). Intracellular superoxide production increased during both RS and dox-induced SIPS

**Sup.Fig. 5** Methylation-specific polymerase chain reaction (PCR) for hTERT

**U)** PCR with unmethylated-specific primers

**M)** PCR with methylated-specific primers

Lanes: **1** and **14** - Molecular Weight Marker (100-1000 bp), **2** - p6 1d, **3** - doxorubicin 1d, **4** - p6 4d, **5** - doxorubicin 4d, **6** - p6 7d, **7** - doxorubicin 7d, **8** - p5, **9** - p10, **10** - p15, **11** - Methylated DNA control, **12** - Unmethylated DNA control, **13** - Control with H_2_O.

*hTERT* promoter was partially methylated during both RS and dox-induced SIPS
